# Interareal Synaptic Inputs Underlying Whisking-Related Activity in the Primary Somatosensory Barrel Cortex

**DOI:** 10.1523/JNEUROSCI.1148-23.2023

**Published:** 2024-01-24

**Authors:** Masahiro Kawatani, Kayo Horio, Mahito Ohkuma, Wan-Ru Li, Takayuki Yamashita

**Affiliations:** ^1^Department of Physiology, Fujita Health University School of Medicine, Toyoake, 470-1192, Japan; ^2^Department of Functional Anatomy and Neuroscience, Graduate School of Medicine, Nagoya University, Nagoya, 466-8550, Japan; ^3^International Center for Brain Science (ICBS), Fujita Health University, Toyoake, 470-1192, Japan

**Keywords:** eOPN3, motor cortex, sensorimotor integration, somatosensory cortex, thalamus, whisker

## Abstract

Body movements influence brain-wide neuronal activities. In the sensory cortex, thalamocortical bottom-up inputs and motor-sensory top-down inputs are thought to affect the dynamics of membrane potentials (*V_m_*) of neurons and change their processing of sensory information during movements. However, direct perturbation of the axons projecting to the sensory cortex from other remote areas during movements has remained unassessed, and therefore the interareal circuits generating motor-related signals in sensory cortices remain unclear. Using a *G_i/o_*-coupled opsin, eOPN3, we here inhibited interareal signals incoming to the whisker primary somatosensory barrel cortex (wS1) of awake male mice and tested their effects on whisking-related changes in neuronal activities in wS1. Spontaneous whisking in air induced the changes in spike rates of a subset of wS1 neurons, which were accompanied by depolarization and substantial reduction of slow-wave oscillatory fluctuations of *V_m_*. Despite an extensive innervation, inhibition of inputs from the whisker primary motor cortex (wM1) to wS1 did not alter the spike rates and *V_m_* dynamics of wS1 neurons during whisking. In contrast, inhibition of axons from the whisker-related thalamus (wTLM) and the whisker secondary somatosensory cortex (wS2) to wS1 largely attenuated the whisking-related supra- and sub-threshold *V_m_* dynamics of wS1 neurons. Notably, silencing inputs from wTLM markedly decreased the modulation depth of whisking phase-tuned neurons in wS1, while inhibiting wS2 inputs did not impact the whisking variable tuning of wS1 neurons. Thus, sensorimotor integration in wS1 during spontaneous whisking is predominantly facilitated by direct synaptic inputs from wTLM and wS2 rather than from wM1.

## Significance Statement

The traditional viewpoint underscores the importance of motor-sensory projections in shaping movement-induced neuronal activity within sensory cortices. However, this study challenges such established views. We reveal that the synaptic inputs from the whisker primary motor cortex do not alter the activity patterns and membrane potential dynamics of neurons in the whisker primary somatosensory cortex (wS1) during spontaneous whisker movements. Furthermore, we make a novel observation that inhibiting inputs from the whisker secondary somatosensory cortex (wS2) substantially curtails movement-related activities in wS1, leaving the tuning to whisking variables unaffected. These findings provoke a reconsideration of the role of motor-sensory projections in sensorimotor integration and bring to light a new function for wS2-to-wS1 projections.

## Introduction

Classical research on anesthetized animals demonstrated that neuronal activity in primary sensory cortices is predominantly shaped by sensory inputs (Mountcastle et al., 1957; Katsuki et al., 1959; Hubel and Wiesel, 1962). However, more recent studies conducted with awake animals have established that spontaneous behaviors such as locomotion and whisking substantially modulate sensory responses of neurons in the primary visual, auditory, and somatosensory areas (Gallant et al., 1998; Crochet and Petersen, 2006; Ferezou et al., 2006; Niell and Stryker, 2010; Polack et al., 2013; Schneider et al., 2014; Zhou et al., 2014; Busse et al., 2017). Although these findings highlight the functional importance of motor-related modulation in the sensory cortex, the neuronal circuit mechanisms underlying these effects are still not fully understood.

The whisker primary somatosensory barrel cortex (wS1) in rodents is among the most extensively studied sensory areas in awake mammalian brains. When mice actively explore their surroundings by rhythmic whisking, wS1 layer 2/3 (L2/3) excitatory neurons slightly depolarize and significantly reduce slow-wave fluctuations of membrane potentials (*V_m_*) (Crochet and Petersen, 2006; Poulet and Petersen, 2008). L2/3 neurons of wS1 exhibit sparse firing properties, and whisking-related signals may not directly influence their firing rates (Crochet and Petersen, 2006; Poulet and Petersen, 2008; Yamashita et al., 2013). However, neurons in deeper layers exhibit higher rates of baseline firing, and spikes of a subset of layer 5 or 6 (L5/6) neurons accurately reflect various variables of whisker movements (Fee et al., 1997; de Kock and Sakmann, 2009; Cheung et al., 2020; Dash et al., 2022). Thalamocortical signals (Poulet et al., 2012), cholinergic modulation (Eggermann et al., 2014; Gasselin et al., 2021), and top-down inputs from the whisker primary motor cortex (wM1) (Lee et al., 2013; Zagha et al., 2013; Naskar et al., 2021) are suggested to play a role in generating supra- and sub-threshold *V_m_* dynamics of wS1 neurons during whisking.

The involvement of interareal inputs in whisking-related activity in wS1 has mainly been inferred from the inhibition of source regions such as the whisker-related thalamic region (wTLM) (Poulet et al., 2012) and wM1 (Xu et al., 2012; Lee et al., 2013). Regional inhibition experiments could involve multiple interconnected brain regions (Otchy et al., 2015). Therefore, direct perturbation of axonal terminals within wS1 would be necessary to examine the role of monosynaptic connections between wS1 and other brain regions. Some previous studies that involve optogenetic stimulation of specific input pathways into sensory cortices revealed the existence of mechanisms that can mimic movement-related state changes (Zagha et al., 2013; Schneider et al., 2014). However, there have been no studies that include specific inhibition of synaptic inputs incoming to primary sensory cortices during motor-related activities. Furthermore, most findings have focused on the superficial layers, leaving the effects of perturbation on the deeper layers largely unexplored.

In this study, we investigate the specific contributions of different inputs to the whisking-related activity of wS1 neurons using awake head-restrained mice. We utilize an inhibitory G protein-coupled opsin, eOPN3, to inactivate axon terminal activity in three regions that provide input to wS1 (Mahn et al., 2021): wTLM, wM1, and the whisker secondary somatosensory cortex (wS2). We first confirmed that photo-inhibition through eOPN3-mediated mechanisms is effective in vitro and in vivo. We then examined the role of each projection to wS1 in the baseline and whisking-induced spiking activities and sub-threshold *V_m_* in wS1 neurons. Our findings suggest that signaling from wM1 to wS1 does not significantly influence the whisking-induced modulation of the activity patterns and *V_m_* dynamics of wS1 neurons. Instead, we demonstrate that signals from wTLM to wS1 and from wS2 to wS1 play significant roles in driving the whisking-induced alterations in the firing rates and *V_m_* dynamics of wS1 neurons. Our results redefine the understanding of sensorimotor integration in wS1 during spontaneous whisking, highlighting the significance of inputs from wTLM and wS2 but not wM1.

## Materials and Methods

### Animals

All procedures for animal experiments followed the guidelines of the Physiological Society of Japan and were approved by the Institutional Animal Care and Use Committee of Fujita Health University. Adult male C57BL6/J mice (2–6 months) were used for experiments. Mice were housed in a temperature-controlled room (23 ± 2°C) with 12 h light/dark cycle (light 0 P.M. to 0 A.M.), with free access to food and water. All experiments using live mice were conducted in the dark phase.

### Viral production

For the production of an adeno-associated virus (AAV) vector introducing eOPN3 expression, HEK293 cells were transfected with a plasmid encoding eOPN3 (pAAV-CaMKIIa(0.4)-eOPN3-mScarlet-WPRE, purchased from Addgene, #125712) together with pHelper and pAAV-RC (serotype 9), using a standard calcium phosphate method. After 3 d, transfected cells were collected and suspended in a lysis buffer (150 mM NaCl, 20 mM Tris pH 8.0). After four freeze–thaw cycles, the cell lysate was treated with 250 U/ml benzonase nuclease (Merck) at 37°C for 10−15 min with adding 1 mM MgCl_2_ and then centrifuged at 4°C at 1,753*×g* for 20 min. AAV was then purified from the supernatant by iodixanol gradient ultracentrifugation. The purified AAV solution was concentrated in PBS via filtration and stored at −80°C.

### Animal preparation and surgery

Mice were anesthetized with isoflurane (2–3% for induction, 1.0–1.5% for maintenance) and head-fixed on a stereotaxic device. Body temperature was maintained at ∼37°C by a controlled heating pad. An ocular ointment was applied over the eyes to prevent drying. AAV-CaMK2a(0.4)-eOPN3-mScarlet-WPRE (the original titer: 4.87 × 10^12^ copies/ml, diluted to 1/2) or AAV-Ef1a-mCherry (#114470-AAV9, obtained from Addgene; titer: 1.0 × 10^13^ copies/ml, diluted to 1/6) were injected into wM1 (AP, +1.0 mm; ML, −1.0 mm; depth, 350 and 850 µm), wTLM (AP, −1.8 mm; ML, −1.8 mm; depth, 3.25 mm) or wS2 (identified by intrinsic imaging; depth, 350 and 850 μm) of the left hemisphere. The total injection volume was 400 nl for wM1, 100−120 nl for wS2, and 200−400 nl for wTLM. After the AAV injection, mice were kept in a home cage for at least 5 weeks before experiments.

Prior to the recording, mice were implanted with a light-weight metal head holder and a recording chamber under isoflurane anesthesia (Yamashita et al., 2013). The chamber was made on the left hemisphere by building a thin wall with dental cement, and the exposed skull was covered by a thin layer of dental cement. We also implanted a small metal screw over the cerebellum as a reference electrode for silicon probe recordings. The locations of the C2 barrel column and wS2 were identified through intrinsic optical imaging (Ferezou et al., 2007; Yamashita et al., 2013) under light isoflurane anesthesia. Before recordings, mice were adapted to head restraint on the recording setup through two or three habituation sessions within 2 or 3 d. In the case of two habituation sessions, the first was 15 min, and the second was 60 min. In the case of three habituation sessions, the first session was 15 min, the second was 30 min, and the third was 60 min. On or one day before the experimental day, a small craniotomy was made over the C2 barrel column. The skull near the craniotomy was thinned for whole-cell recordings to place an optical fiber. When the mice were kept overnight after craniotomy, the exposed brain was protected with a silicon elastomer (Kwik-Cast, WPI). All the whiskers except the left and right C2 were trimmed before experiments.

### Silicon probe recording

Local field potentials (LFP) and extracellular spikes were recorded in head-fixed mice using a silicon probe (A1 × 32-Poly2–10 mm50 s–177, NeuroNexus) with 32 recording sites along a single shank covering 775 µm of the cortical depth (Wang et al., 2020; Li et al., 2023). The probe was stained by dipping it into a DiO solution (1–2% in ethanol) to label the recording site. The probe was then lowered gradually until the tip was positioned at a depth of 800–1,100 µm under the pial surface. The craniotomy site was then covered with Ringer’s solution containing 1.5% agarose, which was further covered by paraffin to avoid desiccation of the solution. Neural data were filtered between 0.1 and 7,603.2 Hz, amplified using a digital head-stage (RHD2132, Intan Technologies), and digitized with a sampling frequency of 30 kHz. The digitized neural signal was transferred to an acquisition board (Open Ephys) and stored on an internal HDD of the host PC for offline analysis. To estimate the depth of L4 in each silicon probe recording, we presented a piezo sensor within reach of the right C2 whisker and recorded LFP upon active whisker touches to the sensor. The TTL pulses for the video acquisition timing, light stimulation, and piezo signals were also recorded through the Open Ephys acquisition board.

### In vivo whole-cell recording

Whole-cell patch-clamp recordings targeted to neurons in the C2 barrel column of awake head-restrained mice were performed using a patch-clamp amplifier (Multiclamp 700B, Molecular Devices) (Yamashita et al., 2013; Yamashita and Petersen, 2016; Wang et al., 2020). Recording pipettes (5–8 MΩ) were pulled from a borosilicate glass capillary and filled with a solution containing (in mM)the following: 135 potassium gluconate, 4 KCl, 10 HEPES, 10 sodium phosphocreatine, 4 Mg-ATP, and 0.3 Na-GTP (pH 7.3, adjusted with KOH, 295 mOsm). The recording pipettes were advanced with positive pressure (20–35 mbar), and a brief negative pressure was applied to establish a giga-ohm seal at encountered cells. The depth of the cells was determined based on the manipulator’s readout that monitors the vertical depth from the pial surface. For the layer definition, we referred to the information provided by a previous report (Lefort et al., 2009). After achieving break-in, step current pulses were applied to monitor the cells’ firing patterns. Only putative pyramidal neurons exhibiting regular spiking patterns were analyzed. All recordings were conducted under the current-clamp configuration. Liquid junction potential was not corrected.

### In vitro whole-cell recording

Mice were transcardially perfused under isoflurane anesthesia with an ice-cold dissection buffer containing (in mM) the following: 87 NaCl, 25 NaHCO_3_, 25 D-glucose, 2.5 KCl, 1.25 NaH_2_PO_4_, 0.5 CaCl_2_, 7 MgCl_2_, and 75 sucrose, aerated with 95% O_2_  + 5% CO_2_. The mice were then decapitated, and the brain was isolated and cut into coronal slices (250–300 µm thick) on a vibratome in the ice-cold dissection buffer. The slices containing wM1 were incubated for 30 min at 35°C in the dissection buffer and maintained thereafter at room temperature (RT) in an artificial cerebrospinal fluid (aCSF) containing (in mM) the following: 125 NaCl, 25 NaHCO_3_, 25 D-glucose, 2.5 KCl, 1.25 NaH_2_PO_4_, 1 MgCl_2_, and 2 CaCl_2_, aerated with 95% O_2_ and 5% CO_2_.

Whole-cell patch-clamp recordings were performed using an IPA amplifier (Sutter Instruments) at RT. Fluorescently labeled cells were visually identified using an upright microscope (BX51WI; Olympus) equipped with a scientific complementary metal-oxide-semiconductor (sCMOS) video camera (Zyla4.2plus, Andor). The recording pipettes were filled with the same intracellular solution used for in vivo whole-cell recording. Patch pipettes (5–7 MΩ) had a series resistance of 6.5–30 MΩ. Data were filtered at 5 kHz, digitized at 10 kHz, and recorded using the SutterPatch software running on Igor Pro 8.

### Filming whisker movement

The mouse orofacial part, including the right C2 whisker, was filmed from a top-view at 500 frames per second using a high-speed camera (HAS-L1, Ditect). Each recording sweep was 60–75 s, synchronized to the electrophysiological recording through TTL pulses.

### Optogenetics

Photo-stimulation during in vivo electrophysiological recordings was conducted by application of green LED light (530 nm; M530F2, Thorlabs) through an fiber optic cannula with a fiber diameter of 400 µm and a numerical aperture of 0.39 (CFML14L05, Thorlabs). The light power at the fiber tip was measured as 7.3 mW. For silicon probe recordings, the tip of the fiber cannula was placed on the brain surface over the recording site. For whole-cell recordings, the tip of the fiber cannula was placed on the thinned skull close to the craniotomy. The green LED was turned on at 5 s before starting a whisker filming epoch and turned off at the end of the epoch. Each epoch was separated for more than 1 min. For silicon probe recordings, we initially collected control data over 10–15 epochs without light illumination (Light OFF condition) and then acquired optogenetic data over 10–15 epochs with light illumination (Light ON condition). For whole-cell recording, data were acquired over 2–6 epochs in the Light OFF condition before collecting an additional 2–6 epochs in the Light ON condition. Light OFF trials consistently preceded Light ON trials due to the long-lasting kinetics of eOPN3 (Mahn et al., 2021). For photo-stimulation during in vitro whole-cell recordings, the green LED light was applied through the objective lens with a light power of 1 mW/mm^2^.

### Data analysis

#### Analysis of whisker movement

Movement of the right C2 whisker was quantified offline with ImageJ using an open-source macro (https://github.com/tarokiritani/WhiskerTracking) (Li et al., 2023). Based on the whisker behavior, recording segments with 1 , 2, or 3 s time windows were classified as a quiet (with no whisker movement) or whisking (with continuous rhythmic whisker movement) epoch. The onset of the whisking epoch was taken as the time the whisker angle exceeded 1° above the baseline.

To decompose whisking bouts, we utilized the previously reported approach (Hill et al., 2011; Sreenivasan et al., 2016). The whisker angular motion θ was broken down into three variables [phase ϕ(*t*), amplitude θ_amp_(*t*), and midpoint θ_mid_(*t*)] at each time point. The whisker angle timeplot was bandpass-filtered within the 4–25 Hz range. The instantaneous phase (ϕ(*t*)) was then determined using the Hilbert transform, where ϕ(*t*) = 0 represents the most protracted position of the whisking cycle and ϕ(*t*) = ± π signifies the end of retraction. Whisking amplitude θ_amp_(*t*) at phases 0 and π was calculated as follows:

$${\rm \theta }_{{\rm amp}\lpar {\rm \phi }\lpar t \rpar = 0\ \amp \ {\rm \phi }\lpar t \rpar ={\pm} \pi \rpar } = \displaystyle{{\lpar {{\rm \theta }_{{\rm \phi }\lpar t \rpar = 0}\ndash {\rm \theta }_{{\rm \phi }\lpar t \rpar ={\pm} \pi }} \rpar } \over 2}.$$

Similarly, whisker midpoint θ_mid_(*t*) at phases 0 and π was defined as follows:

$${\rm \theta }_{{\rm mid}\lpar {\rm \phi }\lpar t \rpar = 0 \ \amp \ {\rm \phi }\lpar t \rpar ={\pm} \pi \rpar } = \displaystyle{{{\rm \theta }_{{\rm \phi }\lpar t \rpar ={\pm} \pi } + \lpar {\rm \theta }_{{\rm \phi }\lpar t \rpar = 0}\ndash {\rm \theta }_{{\rm \phi }\lpar t \rpar ={\pm} \pi }\rpar } \over 2}.$$

Between phases 0 and ± π, amplitude and midpoint values were linearly interpolated. For the whisker motion analysis shown in Figure 13, the whisking setpoint was calculated by applying a low-pass filter to the whisker angle timeplot, with a cutoff frequency of 6 Hz.

#### Analysis of spikes

Spiking activity recorded by a silicon probe was detected and sorted into clusters using Kilosort 2 (https://github.com/MouseLand/Kilosort/releases/tag/v2.0). After an automated clustering step, clusters were manually sorted and refined using Phy 2 (https://github.com/cortex-lab/phy). Well-isolated single units (642 units from wS1 and 55 units from wM1) were included in the dataset. These units were classified as fast-spiking (FS) putative interneurons or regular-spiking (RS) putative pyramidal cells based on their trough-to-peak time of the average spike waveform. Single units with a trough-to-peak time <0.4 ms were classified as FS cells, and units with a trough-to-peak time >0.5 ms were classified as RS cells. Units showing an intermediate (0.4–0.5 ms) trough-to-peak time were excluded from the analysis. For classifying units based on the modulation by whisking, we compared their spike rates during quiet epochs and those during whisking epochs. The units that exhibited significantly higher or lower firing rates during whisking than during quiet epochs were classified as W-Exc or W-Inh units, respectively. The units showing no significant change in their firing rate upon whisking are classified as NM (non-modulated) units. To normalize spike rates for each unit, we obtained the mean and standard deviation (SD) of spike rates of 2-s bins of all filmed sweeps and calculated *Z*-scores. For the presentation of the *Z*-scored peri-event time histogram (PETH) (Figs. 2*C*,*F*, 6*F*, 9*F*), the *Z*-scored spike rates of 200 ms bins were Gaussian filtered and subtracted by the baseline (1.0–0.2 s before the whisking onset).

#### Analyzing tuning curves for whisking variables

Tuning curves for the whisking variables (phase ϕ, amplitude θ_amp_, and midpoint θ_mid_) were computed following methodologies detailed in previous studies (Hill et al., 2011; Sreenivasan et al., 2016; Cheung et al., 2020). We divided the variables into 50 bins, each with equal probability, and computed a histogram of the AP rate for each unit by counting the number of spikes in each bin and dividing by the time duration of that bin. Next, we applied Bayesian adaptive regression splines (BARS) as a smoothing technique to generate the tuning curves from the stimulus–response values (Wallstrom et al., 2008). To evaluate the significance of tuning for each unit, a two-step process was employed, as described previously (Cheung et al., 2020). We first conducted a one-way ANOVA with an α level of 0.05 to determine significant differences in the firing rates across bins. Units that met this criterion were further analyzed; whereby, we shuffled the whisking responses 1,000 times and calculated *F*-values using one-way ANOVA for each iteration. Units were identified as tuned if the observed *F*-value exceeded the 95th percentile of the *F*-value distribution from the shuffled data. We derived maximum and minimum responses from the BARS-fitted tuning curves and calculated the modulation depth as the difference between the maximum and minimum responses, divided by the baseline spike rate. To evaluate the magnitude of tuning, we calculated the modulation depth for each tuning curve, which was defined by the formula (maximal AP rate – minimal AP rate)/baseline AP rate. For displaying polar plots (Figs. 4*C*,*J*, 7*C*, 10*C*), we computed the whisking phase-locked modulation index, defined as (maximal AP rate – minimal AP rate)/(maximal AP rate + minimal AP rate), to facilitate the visualization of the changes in the maximal firing phase of tuned units.

#### Defining layer 4 by current source density analysis

The averaged LFP signals at around active touch contacts were down-sampled to 3 kHz and low-pass filtered at 100 Hz. The LFP signals from odd or even channel numbers were separately used for further analysis. The second spatial derivative of the average LFP signals was used as current source density (CSD) (Freeman and Nicholson, 1975). Sinks were expressed as negative values, and sources were expressed as positive values. The channel with (1) the shortest peak latency for the average touch response in the LFP signal, (2) the largest peak amplitude of the average touch responses in the LFP signal, and (3) the fastest CSD sink onset time upon touches, across 16 channels (the channels either with odd or even numbers among 32 channels), was identified as a center of L4 (Pala and Stanley, 2022), which was estimated as at a subpial depth of 500 µm. Layer boundaries were defined using values reported previously (Lefort et al., 2009).

#### Analysis of LFP power spectrum

The LFP signals captured by a silicon probe electrode situated in L5 were utilized for the analysis. The signals underwent down-sampling to a rate of 1 kHz and were subjected to a low-pass filter with a cutoff frequency of 100 Hz. Epochs classified as either whisking or quiet wakefulness, lasting for more than 2 s, were chosen for further evaluation. Power spectra were computed using the pspectrum function of MATLAB for 2 s segments of the recordings. To evaluate the effect of photo-stimulation (Figs. 2*C*,*D*, 6*C*, 9*C*), the LFP power spectra were normalized to the peak value of the averaged LFP power spectrum obtained in the Light OFF condition.

#### Analysis of membrane potentials

For whole-cell recording data, the mean and variance of *V_m_* and average baseline AP rates were calculated in each whisking or quiet epoch. These values were then averaged across either Light ON or Light OFF trial conditions. In control experiments, where eOPN3 expression was not introduced, we observed that the whisking-associated *V_m_* dynamics in Light ON trials did not statistically differ from those in Light OFF trials (Table 1). We did not utilize the *Z*-score to analyze AP rates because our dataset from in vivo whole-cell recordings included cells that did not exhibit spikes during the recording sessions. Fast Fourier transforms (FFTs) were computed as magnitudes for 2 s segments of the recordings. The amplitude of low-frequency *V_m_* fluctuations was calculated by integrating the computed FFT at 1–5 Hz. To assess *V_m_* modulation in relation to the whisking phase, we aligned the *V_m_* traces as a function of the whisking phase and calculated their average. The modulation depth was subsequently determined as the peak-to-trough difference of this averaged *V_m_* trace. To evaluate the significance of whisking phase-locked modulation for each cell, we employed circular shuffling of individual *V_m_* traces using the circshift function in MATLAB, from which we determined the modulation depth of their average. After completing 1,000 repetitions, we compared the observed modulation depth to the distribution of modulation depths from the shuffled data. The *p*-value represents the fraction of shuffled depths greater than or equal to the observed depth.

**Table 1.** Photo-illumination to wS1 in control mice without eOPN3 expression did not change whisking-induced *V_m_* changes

		*n*	Average	Min.	Max.	SEM	*p* value
Δ*V_m_* (mV)	OFF	9	3.0	0.4	6.6	0.7	0.73
	ON	9	3.3	1.0	6.6	0.6	
ΔAP (Hz)	OFF	9	0.25	−0.13	2.23	0.23	0.44
	ON	9	−0.022	−0.16	0.021	0.017	
ΔVariance (µV^2^)	OFF	9	−16.3	−38.6	21.9	6.4	0.50
	ON	9	−14.2	−30.5	15.4	4.8	
Whisking phase-locked	OFF	3	1.6	1.0	2.5	0.4	0.75
*V_m_* amplitude (mV)	ON	3	1.3	0.7	1.7	0.2	

OFF: Light OFF condition, ON: Light ON condition. Wilcoxon signed rank test (Light OFF vs Light ON).

### Statistics

All values are expressed as box plots in the figures. The edges of the box indicate 25th and 75th percentiles, and the central line indicates median value. The whiskers extend to the most extreme data points, excluding outliers. Statistical tests were performed using MATLAB or GraphPad Prism. The normality of data distribution was routinely tested. Analyses of two-sample comparisons were performed using paired *t* tests, when each sample was normally distributed, or using Wilcoxon rank sum (unpaired) or Wilcoxon signed rank (paired) test when at least one of the samples in every two-sample comparison was not normally distributed. When we performed multiple two-sample comparisons for the classification of W-Exc, W-Inh, and NM units, *p*-values were corrected using the Benjamini–Hochberg procedure to control the false discovery rate (Benjamini and Hochberg, 1995), unless otherwise noted. Analyses of one-sample comparisons were performed using one-sample *t* tests when each sample was normally distributed, or Wilcoxon signed rank test when the samples were not normally distributed. Statistical analyses for comparisons of LFP power spectra were carried out using two-way ANOVA.

## Results

### Impact of motor-sensory signaling on wS1 activity

**Figure 1.**
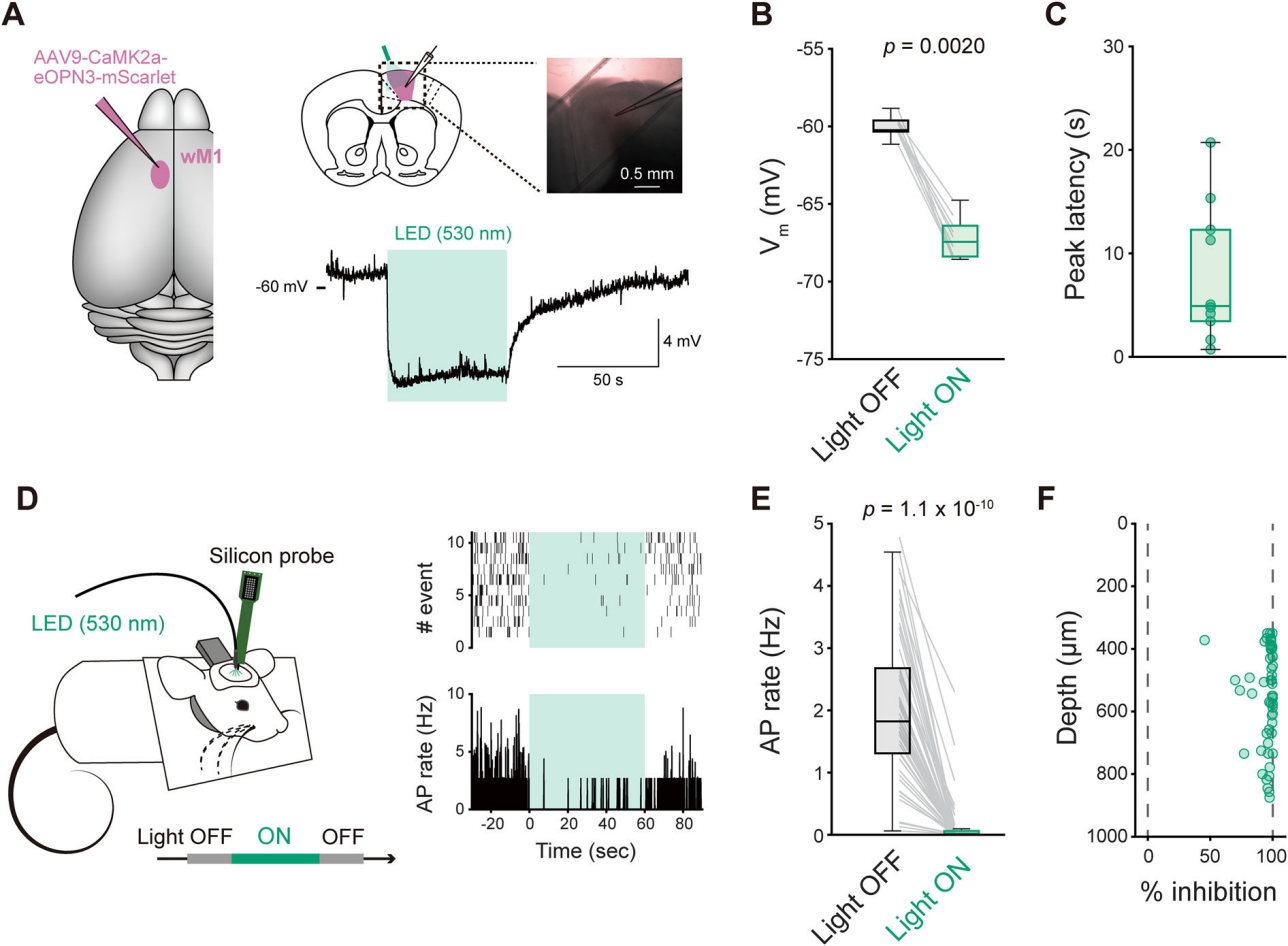
Inhibitory effect of light-induced activation of eOPN3 on neuronal membrane potentials and firings. ***A***, Left: schematic of viral injection. Right: schematic of in vitro patch-clamp recordings (top), a bright field image of a brain slice during recording (inset) and a representative *V_m_* trace upon green light illumination (bottom). Purple: eOPN3-mScarlet, green shadow: photo-stimulation. ***B***, Averaged *V_m_* with (Light ON) or without (Light OFF) photo-stimulation. ***C***, Peak latencies of the responses to the photo-stimulation. ***D***, Left: Schematic of silicon probe recording from wM1 using an awake head-restrained mouse. Right: Example raster plot obtained from a representative wM1 unit (top) and corresponding peri-stimulus time histogram upon green light illumination onto wM1 (green shadow). ***E***, Baseline AP rates of wM1 units with (Light ON) or without (Light OFF) green light illumination onto wM1 in vivo. ***F***, Percentage of the inhibition of baseline AP generation in wM1 plotted as a function of recording depth. Gray lines (***B***, ***E***) and circles (***C***, ***F***) indicate individual cells/units. The *p*-values are indicated in the figure panels. Wilcoxon signed rank test (***B***, ***E***).

Neuronal activity in wM1 can influence wS1 activity (Xu et al., 2012; Lee et al., 2013; Pais-Vieira et al., 2013; Zhang and Zagha, 2023). This impact can either originate directly from axonal terminals of wM1 neurons that project to wS1 (Petreanu et al., 2009, 2012; Lee et al., 2013; Zagha et al., 2013; Kinnischtzke et al., 2014; Naskar et al., 2021) or indirectly through signaling from other brain areas (Urbain and Deschênes, 2007b; Pais-Vieira et al., 2013). To elucidate the circuit mechanisms underlying wM1 → wS1 signaling, we employed a *G_i__/o_*-coupled inhibitory opsin, eOPN3 (Mahn et al., 2021), for optogenetic inhibition of ipsilateral wM1 or wM1 axonal projections in wS1 (wM1 → wS1 axons). We virally introduced expression of eOPN3 in wM1 neurons and used the mice after a minimum of 5 weeks following the viral injection. We first evaluated the inhibitory effect of light-induced activation of eOPN3 on neuronal activity. In acute brain slice preparations, green LED illumination (530 nm, 1.0 mW/mm²) induced a robust hyperpolarization in eOPN3-expressing wM1 neurons (Fig. 1*A,B*; −7.2 ± 0.4 mV, *n* = 10 cells) with a mean peak latency of 8.0 ± 2.0 s (Fig. 1*C*; *n* = 10 cells). In awake head-restrained mice, green light exposure (530 nm, 7.3 mW at the fiber tip) applied to the surface of wM1 markedly reduced action potential (AP) rates of wM1 cells as recorded by a silicon probe (Fig. 1*D*–*E*; by 95.1 ± 1.3%, *n* = 55 units). We observed these photo-inhibitory effects consistently across all cortical layers (Fig. 1*F*), suggesting the efficient inhibition of neural activity in vivo via eOPN3-mediated mechanisms.

**Figure 2.**
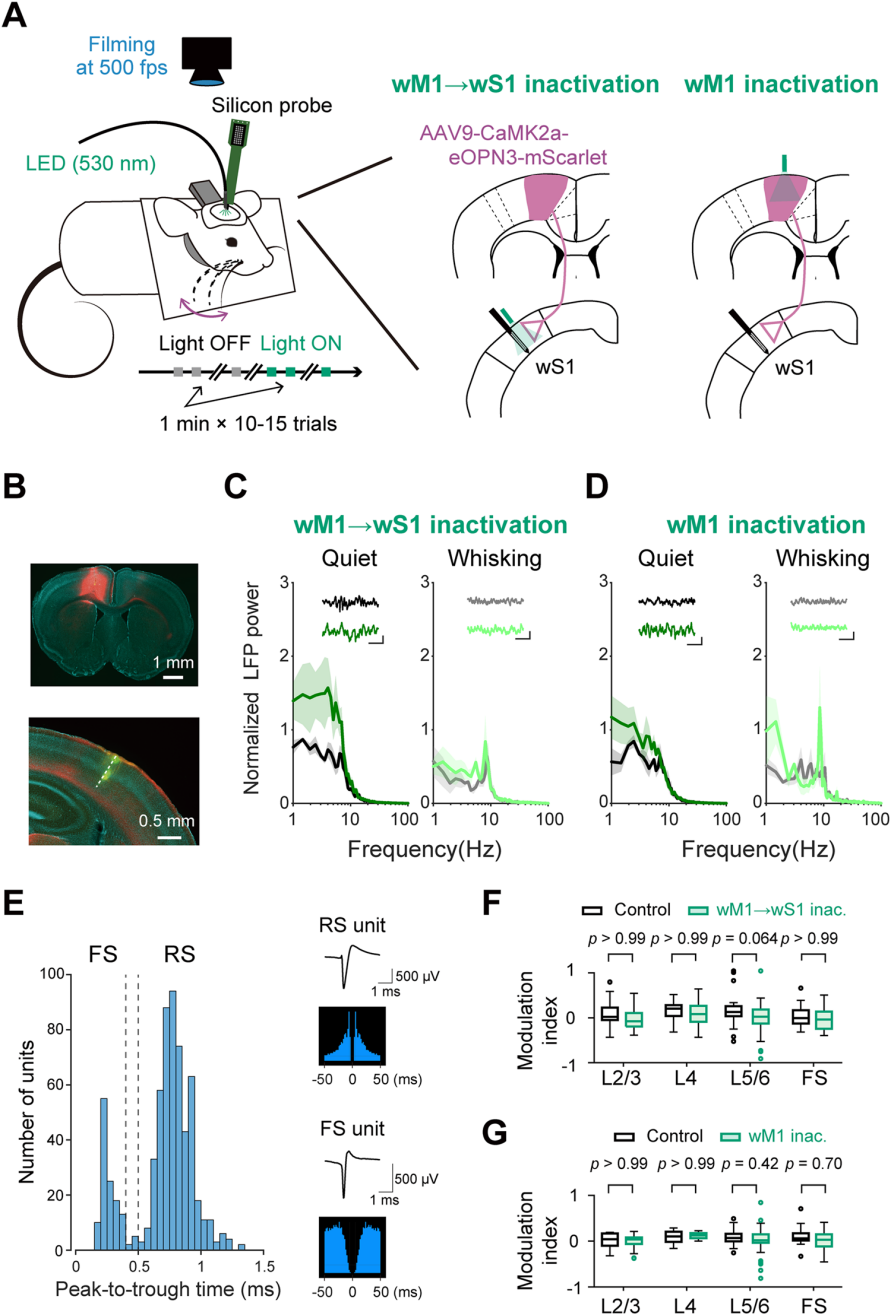
Silicon probe recordings in wS1 with photo-inactivation of ipsilateral wM1 or wM1 → wS1 axons. ***A***, Schematic of the experiment. ***B***, Epifluorescence image of a coronal brain section containing the wM1 injection site (top) or the wS1 recording site (bottom). The dashed line indicates the inserted electrode’s trace. Green: DiO, red: eOPN3-mScarlet, blue: DAPI. ***C****,* Normalized LFP power spectra in wS1 during quiet wakefulness (left) and whisking (right), without (black or gray) or with (green or light green) inactivation of the wM1 → wS1 axons. Insets: LFP traces with (bottom) and without (top) the inactivation. The slow components of LFP signals were modulated by the inactivation (1−5 Hz power; quiet: *F*_(1,37)_ = 29.09, *p* = 2.60 × 10^−6^; whisking, *F*_(1,37)_ = 5.95, *p* = 0.019; two-way ANOVA, Light ON vs Light OFF, *n* = 3 mice). Scale bar: 0.5 s, 0.2 mV; solid line and shading: mean ± SEM. ***D***, Same as ***C***, but for wM1 inactivation (1−5 Hz power; quiet: *F*_(1,37) _= 14.69, *p* = 0.0004; whisking: *F*_(1,37) _= 0.40, *p* = 0.53; two-way ANOVA, Light ON vs Light OFF, *n* = 3 mice). Scale bar: 0.5 s, 0.2 mV. ***E***, Histogram of peak-to-trough time of recorded units in all wS1 recordings. Units are categorized as RS (peak-to-trough time >0.5 ms) or FS (peak-to-trough time <0.4 ms) units. Insets: average AP waveform and auto-correlogram from representative RS (top) and FS (bottom) units. ***F***, Modulation indices of baseline AP rates during quiet wakefulness upon green light illumination onto wS1 in mCherry-expressing mice (Control, 88 units from 2 mice) or eOPN3-expressing mice (wM1 → wS1 inac., 104 units from 3 mice). The modulation index was calculated as (AP rate in Light ON – AP rate in Light OFF)/ (AP rate in Light ON + AP rate in Light OFF). Open circles indicate data points from outliers. ***G***, Same as ***F***, but for photo-stimulation of wM1 (mCherry-expressing mice [Control]: 71 units from 2 mice; eOPN3-expressing mice [wM1 inac.]: 94 units from 2 mice). The *p*-values are indicated in the figure panels. Bonferroni’s multiple comparison test (***F***, ***G***).

We next utilized these mice expressing eOPN3 in wM1 neurons for silicon probe recording from an identified left C2 barrel column in wS1 while either ipsilateral wM1 or wM1 → wS1 axons in wS1 were photo-inhibited using green light (Fig. 2*A*,*B*). The mice were awake and head-restrained, and we filmed whisker movements at a rate of 500 Hz using a high-speed camera to compare non-whisking quiet wakefulness and the whisking state. We first attempted to examine the overall network effect of the photo-inhibition by measuring the LFP recorded at wS1 L5. Upon photo-inhibition of either wM1 → wS1 axons in wS1 or wM1 neurons, the slow components of the LFP power at 1−5 Hz were enhanced during quiet wakefulness (Fig. 2*C*,*D*). These results indicate that the direct or indirect synaptic inputs signaled from wM1 can affect wS1 network status, consistent with a previous report (Zagha et al., 2013).

**Figure 3.**
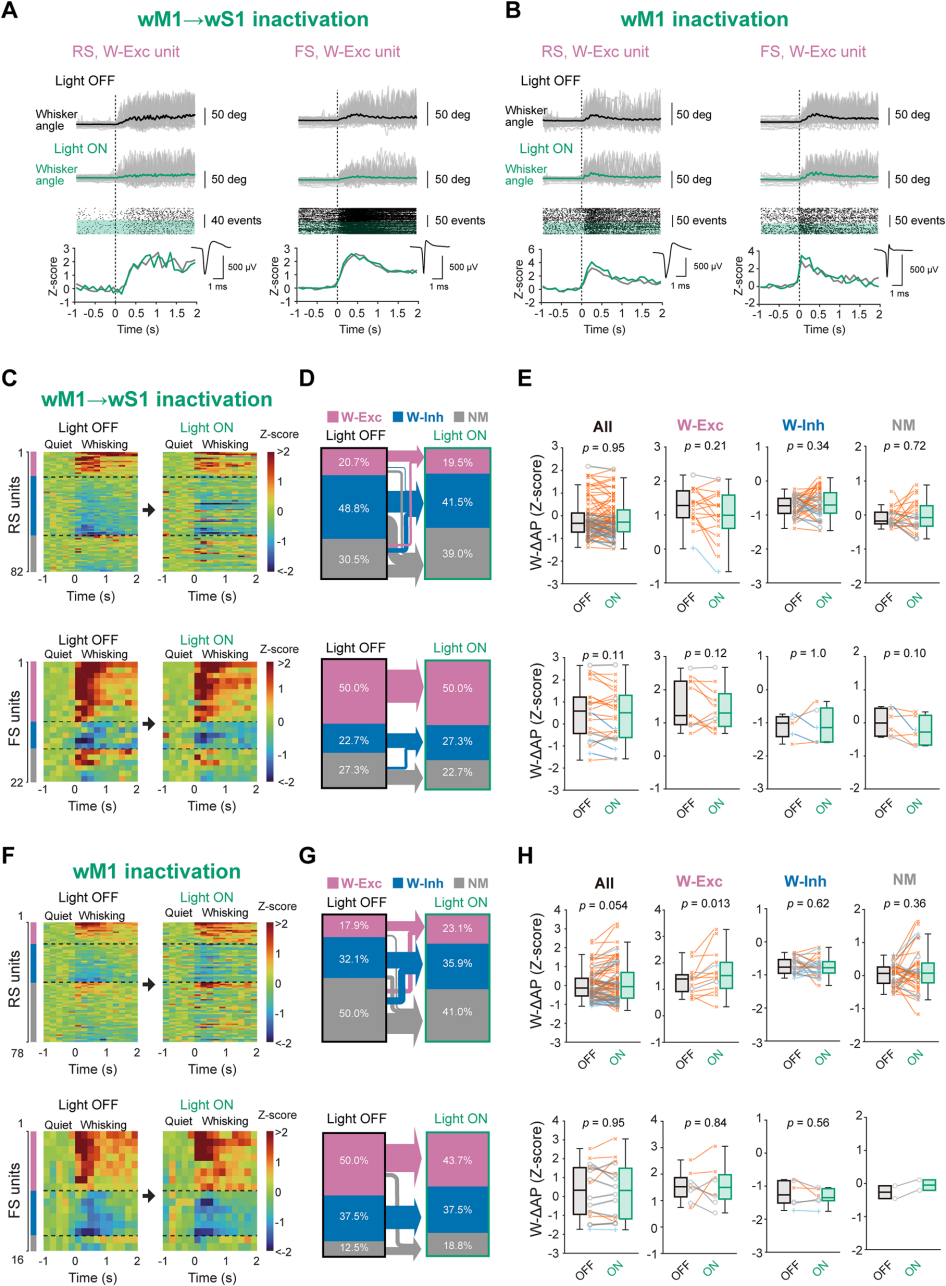
No effects of inactivation of wM1 or wM1 → wS1 axons on whisking-related activity in wS1. ***A***, Example whisker angle timeplots (top), raster plots (middle), and *Z*-scored PETH (bottom) aligned to the whisking onset with (Light ON) or without (Light OFF) the photo-inhibition of wM1 → wS1 axons from representative RS (left) and FS (right) units. Small insets on the bottom show averaged AP waveforms of the corresponding units. Green shading in the raster plot indicates Light ON trials. ***B***, Same as ***A***, but with wM1 inactivation. ***C***, The *Z*-scored PETHs of individual RS (top) and FS (bottom) units (200 ms bin size) aligned to the whisking onset with (Light ON) and without (Light OFF) inactivation of the wM1 → wS1 axons. ***D***, Proportions of whisking-modulated RS (top) or FS (bottom) units in wS1 with (Light ON) and without (Light OFF) the wM1 → wS1 inactivation, categorized into three groups: positively modulated units (W-Exc, purple), negatively modulated units (W-Inh, blue), and non-modulated units (NM, gray). Arrows show transitions of the cellular categories from Light OFF to Light ON trials. ***E***, Whisking-related changes in the *Z*-scored AP rates (W-ΔAP) of RS (top) and FS (bottom) units with (Light ON) and without (Light OFF) the wM1 → wS1 inactivation. ***F***−***H***, Same as ***C***−***E***, but for wM1 inactivation. Blue lines and crosses: L2/3 units, gray lines and circles: L4 units, orange lines and saltires: L5/6 units. The *p*-values are indicated in the figure panels. Wilcoxon signed rank test (***E***: RS All, RS W-Exc, RS W-Inh, RS NM, FS W-Exc and FS W-Inh units, ***H***: RS W-Exc, RS W-Inh, RS NM, FS W-Inh and FS W-Exc units), or paired t test (***E***: FS All and FS NM units, ***H***: FS All units).

We next analyzed how the motor-sensory signaling affects AP rates of individual units. We sorted well-separated single units and categorized them into two groups: regular-spiking (RS) and fast-spiking (FS) neurons, depending on the peak-to-trough time of their AP waveform (Fig. 2*E*). As control experiments, we virally introduced mCherry expression in wM1 and recorded from ipsilateral wS1 while illuminating wM1 (the viral injection site) or wS1 (the recording site) with green light. During quiet wakefulness, neither photo-inhibition of wM1 → wS1 axons nor that of wM1 neurons resulted in significant changes in the overall baseline AP rate of RS and FS units in wS1 compared to the corresponding control groups (Fig. 2*F*,*G*). We further examined whether the motor-sensory signaling might affect the whisking-related neural activity in wS1 (Fig. 3*A*,*B*). Among 82 RS units we studied, whisking increased AP rates in 17 units (20.7%, termed W-Exc) without any optogenetic perturbation (Light OFF condition; Fig. 3*C*,*D*). In contrast, 40 (48.8%) of RS units exhibited a decrease (W-Inh), and 25 (30.5%) showed no changes (non-modulated [NM]) in AP rates during whisking (Fig. 3*C,D*). When we photo-inhibited wM1 → wS1 axons with eOPN3 (Light ON condition), 23.5% (4 out of 17) of W-Exc and 32.5% (13 out of 40) of W-Inh RS units lost their responsiveness to whisking (Fig. 3C*,D*). However, the whisking-related changes in the *Z*-scored AP rates (W-ΔAP) in all units studied or each of these three categories were not significantly affected by photo-inhibition of wM1 → wS1 axons (Fig. 3*E*; All: Light OFF −0.14 ± 0.09, Light ON −0.13 ± 0.09, *p* = 0.95; W-Exc: Light OFF 1.23 ± 0.13, Light ON 0.99 ± 0.18, *p* = 0.21; W-Inh: Light OFF −0.74 ± 0.04 Light ON −0.66 ± 0.06, *p* = 0.34; NM: Light OFF 0.09 ± 0.05, Light ON 0.05 ± 0.08, *p* = 0.72; Wilcoxon signed rank test for all four comparisons). We also analyzed FS units (*n* = 22 units from three mice). Our recordings identified 11 (50.0%) W-Exc, 5 (22.7%) W-Inh, and 6 (27.3%) NM units among FS units (Fig. 3*C*,*D*). Most of FS units did not change their category under photo-inhibition of wM1 → wS1 axons (Fig. 3*C*,*D*). The axonal inhibition did not significantly impact the W-ΔAP of FS units (Fig. 3*E*; All: Light OFF 0.55 ± 0.27, Light ON 0.42 ± 0.25, *p* = 0.11; W-Exc: Light OFF 1.60 ± 0.21, Light ON 1.45 ± 0.18, *p* = 0.12; W-Inh: Light OFF −1.11 ± 0.15 Light ON −1.05 ± 0.22, *p* = 1.0; NM: Light OFF 0.02 ± 0.15, Light ON −0.22 ± 0.16, *p* = 0.10; paired *t* test for All and NM comparisons, and Wilcoxon signed rank test for W-Exc and W-Inh comparisons). These results suggest that wM1 → wS1 inputs do not substantially affect the overall activity patterns of wS1 neurons during whisking, although they could influence the whisking responsiveness of a subset of RS neurons within wS1.

We also examined the effect of photo-inhibition of ipsilateral wM1 on the whisking-induced modulation of neuronal activities in wS1 (Fig. 3*B*). The wM1 photo-inhibition led to an increase in the W-ΔAP in W-Exc RS units (Light OFF 1.34 ± 0.14, Light ON 1.64 ± 0.21, *n* = 14 units, *p* = 0.013, Wilcoxon signed rank test) and promoted a slight increase in the proportion of W-Exc units by recruiting a certain fraction of (6 out of 39) NM RS units (Fig. 3*F*–*H*). While photo-inhibition of wM1 seemed to recruit some (8 out of 39) NM units to be W-Inh units, the W-ΔAP in W-Inh RS units remained unchanged by light (Fig. 3*F–H*; Light OFF −0.73 ± 0.05, Light ON −0.75 ± 0.06, *n* = 25 units, *p* = 0.62, Wilcoxon signed rank test). No significant effects of wM1 photo-inhibition on FS units were observed (Fig. 3*F*–*H*). These findings suggest that wM1 activity may suppress whisking-evoked spiking in a subset of wS1 neurons. However, wM1 → wS1 axons do not appear to mediate this effect, suggesting the involvement of different circuit mechanisms potentially including indirect pathways.

**Figure 4.**
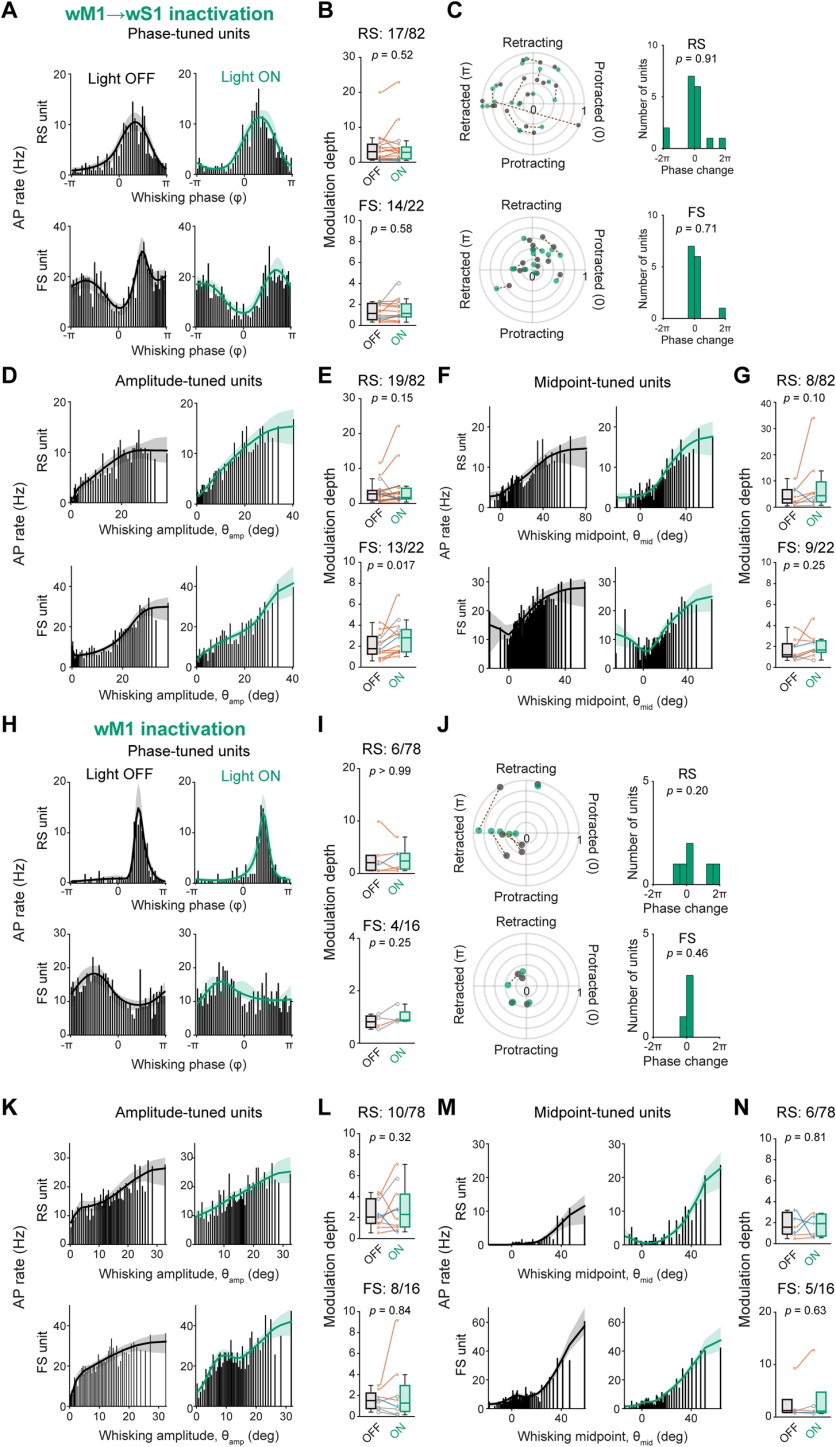
Little effects of inactivation of wM1 or wM1 → wS1 axons on whisking variable-tuning of wS1 neurons. ***A***, Example RS (top) and FS (bottom) units tuned to the whisking phase. The histograms and tuning curves of the example units with (Light ON, right) and without (Light OFF, left) inactivation of wM1 → wS1 axons were plotted. ***B***, Normalized modulation depth in phase-tuned units with (ON) and without (OFF) the wM1 → S1 inactivation. The modulation depth was calculated as the difference between maximum and minimum AP rate within the whisking cycle, and divided by the value with baseline AP rate for normalization. Numbers of tuned units among recorded units are indicated. ***C***, Left, polar plots showing the peak-normalized modulation depth vs maximal-firing phase in the whisk cycle for RS (top) or FS (bottom) units with (green) and without (black) the wM1 → wS1 inactivation. Dotted lines connect data from identical units. Right, distributions of the change in the maximal firing phase for the tuned units. ***D***, ***E***, Same as (***A***, ***B***), but for amplitude-tuned units. ***F***, ***G***, Same as (***A***, ***B***), but for midpoint-tuned units. ***H***−***N***, Same as (***A***−***G***), but with wM1 inactivation. Shadings in tuning curves (in ***A***, ***D***, ***F***, ***H***, ***K***, and ***M***) indicate 95% confidence intervals. The *p*-values are indicated in the figure panels. Two-sample (***B, E, G, I, L, N***) or one-sample (***C***, ***J***, vs 0) Wilcoxon signed rank test.

Previous studies have identified specific subsets of wS1 neurons that represent various aspects of whisker movements, including the whisking phase as well as the amplitude (θ_amp_) and midpoint (θ_mid_) of the whisker positioning (Fee et al., 1997; Szwed et al., 2003; Curtis and Kleinfeld, 2009; Cheung et al., 2020). The wM1 also contains neurons showing such tuning of the whisking-related variables (Hill et al., 2011; Sreenivasan et al., 2016). Therefore, we next analyzed the effects of photo-inhibition of the motor-sensory signaling on the degree of neuronal tuning (Fig. 4). We found that subsets of RS and FS units in wS1 display notable tuning to whisking phase, θ_amp_, and θ_mid_. Photo-inhibition of either wM1 → wS1 axons (Fig. 4*A–G*) or ipsilateral wM1 (Fig. 4*H–N*) did not result in any significant changes in their modulation depths, except that the θ_amp_ tuning by FS units was slightly enhanced by the wM1 → wS1 inactivation (Fig. 4*D*,*E*; modulation depth for θ_amp_ tuning: Light OFF 2.07 ± 0.30, Light ON 2.82 ± 0.44, *n* = 13 units, *p* = 0.017, Wilcoxon signed rank test). These results suggest that motor-sensory signaling does not substantially influence the tuning of whisking variables in most of the tuned units in wS1.

**Figure 5.**
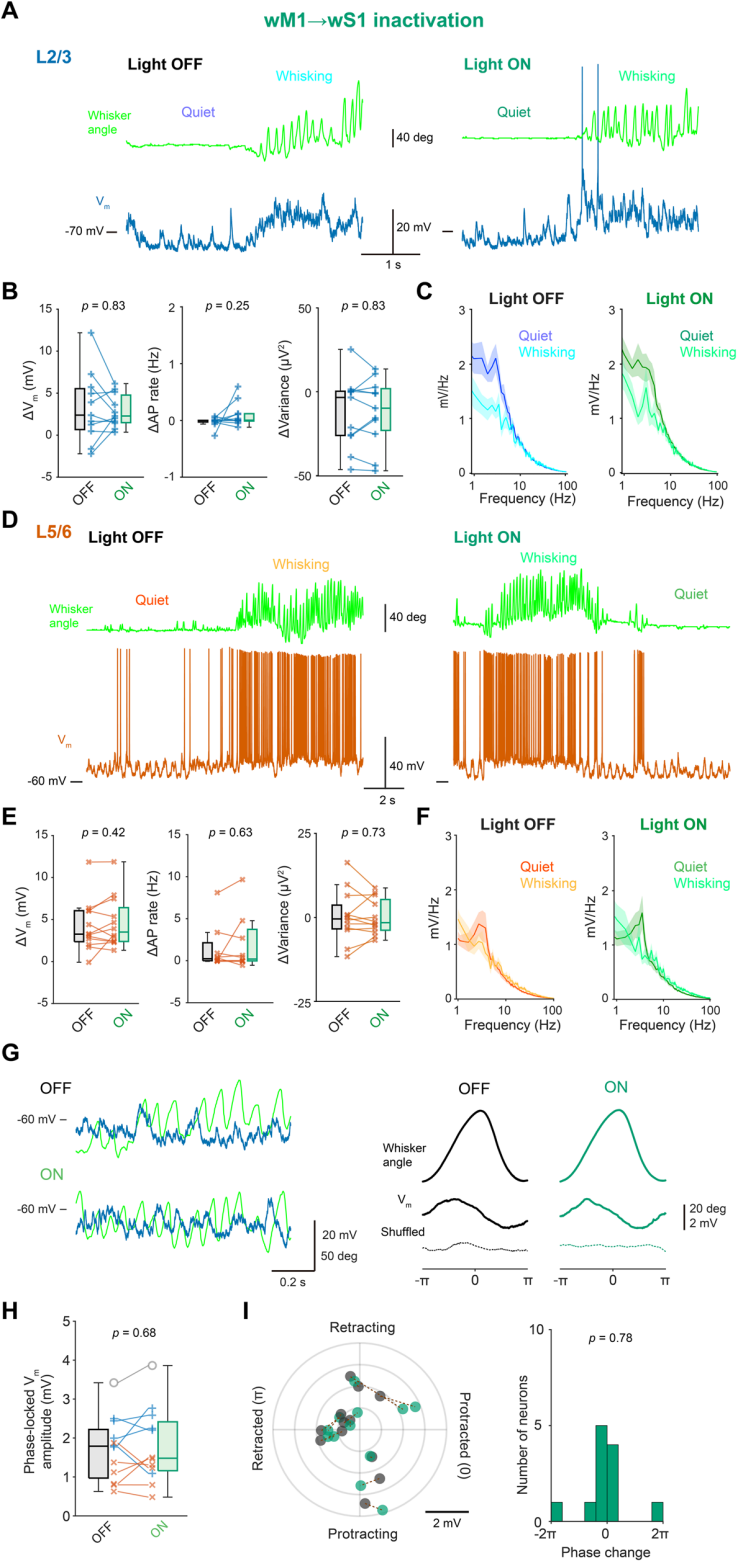
No effects of wM1 → wS1 inactivation on the membrane potential of wS1 neurons. ***A***, Example membrane potential recording from a neuron at L2/3 of wS1 with (Light ON) and without (Light OFF) inactivation of the wM1 → wS1 axons. ***B***, Whisking-induced changes in mean *V_m_* (left), AP rate (middle), and *V_m_* variance (right) of L2/3 wS1 neurons with (ON) and without (OFF) wM1 → wS1 inactivation. ***C***, Averaged *V_m_* FFT during quiet wakefulness and whisking (*n* = 10 cells). ***D***−***F***, Same as (***A***–***C***), but from L5/6 neurons of wS1 (*n* = 11 cells). ***G***, Left, example *V_m_* recording during whisking from an L2/3 neuron in wS1 with (ON) and without (OFF) wM1 → wS1 inactivation. Right, averaged whisker angle (top), averaged *V_m_* traces (middle), and shuffled *V_m_* averages (bottom) as a function of whisking phase, obtained from the example neuron. ***H***, Amplitudes of whisking phase-locked *V_m_* fluctuation with (ON) and without (OFF) wM1 → wS1 inactivation. ***I***, Left, the magnitude of phase-locked *V_m_* fluctuation and the most depolarized phase with (green) and without (black) wM1 → wS1 inactivation. Dotted lines connect data from identical cells. Right, the distribution of shifts in the most depolarized phase. Solid line and shading (in ***C, F***) represent mean ± SEM. The *p*-values are indicated in the figure panels. Wilcoxon signed rank test (***B***, ***E***), one-sample *t* test vs 0 (***I***).

The analysis of spikes recorded with extracellular electrodes often exhibits a bias toward cells with relatively higher spike rates (Shoham et al., 2006; Barth and Poulet, 2012). This bias is especially significant in superficial layers of wS1, which contain many cells with very low spike rates that are typically overlooked by spike analyses. We therefore examined the effects of photo-inhibition of wM1 → wS1 axons on sub-threshold *V_m_* dynamics in wS1 neurons associated with spontaneous whisking. Using in vivo whole-cell recordings, we recorded from RS neurons in L2/3 or L5/6 of wS1 (Fig. 5). Photo-inhibition of wM1 → wS1 axons did not change resting *V_m_* and baseline AP rate during quiet wakefulness (Table 2). The wM1 → wS1 photo-inhibition also did not significantly affect *V_m_* depolarization, AP rate changes, or the changes in *V_m_* fluctuation upon whisking in L2/3 or L5/6 RS neurons (Fig. 5*A*–*F*). Even in eight neurons, which exhibited an increase of AP rates by more than 0.1 Hz during whisking, photo-inhibition of wM1 → wS1 axons did not affect the magnitude of *V_m_* depolarization and AP rate change upon whisking (*V_m_* depolarization: Light OFF 4.2 ± 0.8 mV, Light ON 4.9 ± 0.7 mV, *p* = 0. 313, Wilcoxon signed rank test; ΔAP rate: Light OFF 3.6 ± 1.8 Hz, Light ON 5.4 ± 2.9 Hz, *p* = 0.461, Wilcoxon signed rank test).

**Table 2.** Effects of photo-inhibition of wM1/wTLM/wS2 → wS1 axons on baseline AP rate and mean *V_m_* during quiet wakefulness

				*n*	Average	Min.	Max.	SEM	*p* value
wM1	AP rate (Hz)	All	OFF	23	0.68	0.00	7.21	0.37	0.53
			ON	23	0.62	0.00	7.01	0.32	
		L2/3	OFF	10	0.03	0.00	0.15	0.01	0.22
			ON	10	0.05	0.00	0.19	0.02	
		L5/6	OFF	12	1.27	0.00	7.21	0.66	0.92
			ON	12	1.14	0.00	7.01	0.57	
	*V_m_* (mV)	All	OFF	23	−63.1	−79.7	−43.2	2.0	0.064
			ON	23	−62.0	−76.0	−46.7	1.8	
		L2/3	OFF	10	−62.2	−79.7	−51.3	2.8	0.29
			ON	10	−60.6	−74.9	−49.1	2.3	
		L5/6	OFF	12	−62.7	−74.2	−43.2	2.7	0.47
			ON	12	−62.0	−75.9	−46.7	2.5	
wTLM	AP rate (Hz)	All	OFF	27	0.53	0.00	5.54	0.22	0.37
			ON	27	0.75	0.00	8.21	0.35	
		L2/3	OFF	14	0.23	0.00	1.78	0.13	0.63
			ON	14	0.14	0.00	0.60	0.05	
		L5/6	OFF	13	0.85	0.00	5.54	0.41	0.27
			ON	13	1.41	0.00	8.21	0.68	
	*V*_*m*_ (mV)**	All	OFF	27	−64.5	−83.1	−44.3	2.1	0.00015
			ON	27	−61.7	−79.5	−42.4	2.0	
		L2/3	OFF	14	−71.5	−83.1	−57.4	1.9	0.00061
			ON	14	−68.1	−79.5	−57.3	2.0	
		L5/6	OFF	13	−56.9	−80.4	−44.3	2.5	0.02
			ON	13	−54.9	−78.6	−42.4	2.4	
wS2	AP rate (Hz)	All	OFF	25	0.39	0.00	1.98	0.12	0.52
			ON	25	0.45	0.00	3.18	0.16	
		L2/3	OFF	13	0.12	0.00	1.24	0.09	0.95
			ON	13	0.11	0.00	0.67	0.06	
		L5/6	OFF	10	0.76	0.00	1.98	0.23	0.50
			ON	10	0.92	0.00	3.18	0.33	
	*V_m_* (mV)	All	OFF	25	−66.5	−83.9	−46.2	2.1	0.19
			ON	25	−65.3	−83.5	−45.5	2.1	
		L2/3	OFF	13	−72.8	−83.9	−57.5	2.4	0.24
			ON	13	−71.4	−83.5	−56.5	2.4	
		L5/6	OFF	10	−57.5	−66.6	−46.2	1.7	0.50
			ON	10	−56.8	−72.4	−45.5	2.3	

OFF: Light OFF condition, ON: Light ON condition. Wilcoxon signed rank test (Light OFF vs Light ON).

A subset of pyramidal neurons in wS1 shows rapid *V_m_* dynamics phase-locked to the whisking cycle (Crochet and Petersen, 2006; Poulet and Petersen, 2008; Yamashita et al., 2013). We therefore analyzed the phase-locked *V_m_* modulation of neurons, but it was found not to be affected by photo-inhibition of wM1 → wS1 axons (Fig. 5*G*–*I*; Light OFF 1.7 ± 0.3 mV, Light ON 1.8 ± 0.3 mV, *p* = 0.68, *n* = 12 cells). Together with the results of extracellular recordings, our results thus argue against the role of wM1 → wS1 signaling in the whisking-related activity of wS1, instead suggesting that the motor-sensory projection may not significantly alter the activity patterns and *V_m_* dynamics of wS1 neurons during spontaneous whisking.

### Influence of thalamocortical signaling on wS1 activity during quiet and whisking states

**Figure 6.**
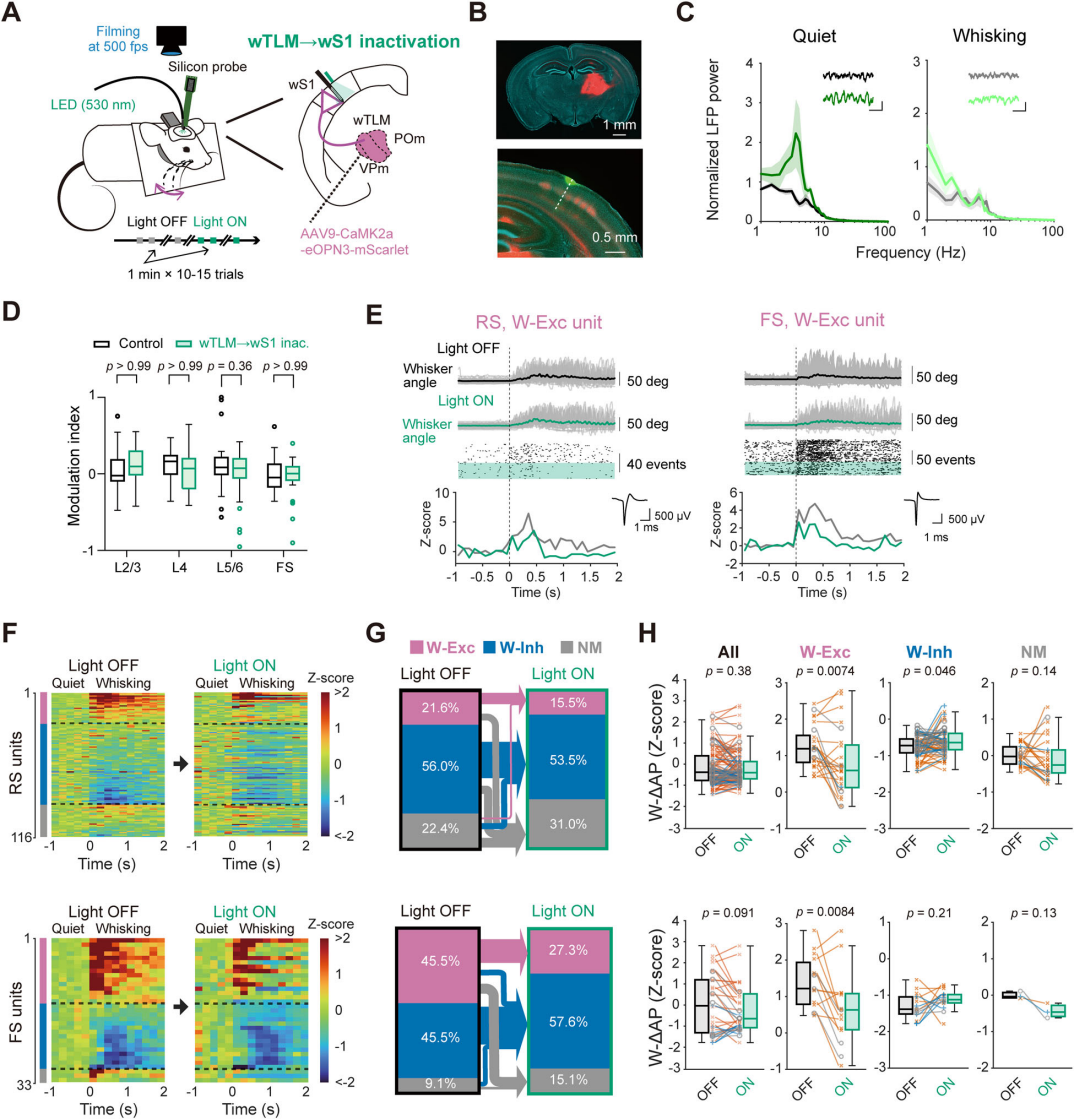
Effects of inactivation of wTLM → wS1 axons on neuronal activity in wS1. ***A***, Schematic of the experiment. ***B***, Epifluorescence image of a coronal brain section containing the wTLM injection site (top) or the wS1 recording site (bottom). The dashed line indicates the inserted electrode’s trace. Green: DiO, red: eOPN3-mScarlet, blue: DAPI. ***C****,* Normalized LFP power spectra in wS1 during quiet wakefulness (left) and whisking (right), with (green or light green) or without (black or gray) inactivation of wTLM → wS1 axons. Insets: representative LFP traces with (bottom) and without (top) inactivation. The inactivation modulated the slow components of LFP signals (1−5 Hz power; quiet: *F*_(1,55)_ = 18.71, *p *= 5.27 × 10^−5^ ; whisking: *F*_(1,55)_ = 6.18, *p* = 0.015; two-way ANOVA, Light ON vs Light OFF, *n* = 4 mice). Scale bar: 0.5 s, 0.2 mV; solid line and shading: mean ± SEM. ***D***, Modulation indices of baseline AP rates during quiet wakefulness upon green light illumination onto wS1 in control mice (same data as in Fig. 2*F*) or eOPN3-expressing mice (wTLM → wS1 inact., 149 units from 3 mice). Open circles indicate data points from outliers. ***E***, Example whisker angle timeplots (top), raster plots (middle), and *Z*-scored PETH (bottom) aligned to the whisking onset with (Light ON) or without (Light OFF) inhibition of wTLM → wS1 axons from representative RS (left) and FS (right) units. Small insets on the bottom show averaged AP waveforms of the corresponding units. Green shading in the raster plot indicates Light ON trials. ***F***, *Z*-scored PETHs of individual RS (top) and FS (bottom) units (200 ms bin size) aligned to the whisking onset with (Light ON) and without (Light OFF) inactivation of the wTLM → wS1 axons. ***G***, Proportions of whisking-modulated RS (top) or FS (bottom) units in wS1 with (Light ON) and without (Light OFF) the wTLM → wS1 inactivation, separated into the W-Exc, W-Inh, and NM categories. Arrows show transitions of the cellular categories. ***H***, W-ΔAP (in *Z*-score) of RS (top) and FS (bottom) units with (Light ON) and without (Light OFF) the wTLM → wS1 inactivation. Blue lines and crosses: L2/3 units, gray lines and circles: L4 units, orange lines and saltires: L5/6 units. The *p*-values are indicated in the figure panels. Bonferroni’s multiple comparison test (***D***), Wilcoxon signed rank test (***H***, RS All, RS W-Exc, RS W-Inh, FS All, and FS W-Exc units), or paired *t* test (***H***, RS NM, FS W-Inh, and FS NM units).

Whisking induces a substantial increase in the AP rate of wTLM neurons (Poulet et al., 2012; Urbain et al., 2015; Petty et al., 2021). Such whisking-related activity of wTLM is known to elicit depolarization of wS1 neurons (Poulet et al., 2012) and may thus contribute to whisking-related wS1 spiking activity. However, wTLM has a broad spectrum of projections to the cerebral cortex, including wS1, wM1, and wS2 (Deschênes et al., 1998; Urbain and Deschênes, 2007a; Wimmer et al., 2010; El-Boustani et al., 2020), making unclear which projections mainly affect wS1 circuits during whisking. To clarify the role of wTLM → wS1 monosynaptic inputs in wS1 activity, we conducted photo-inhibition of the wTLM → wS1 axons using eOPN3 (Fig. 6*A,B*). We first analyzed the effect of the photo-inhibition on the LFP power at L5 and found the slow-wave components at 1−5 Hz were well increased upon photo-inhibition, both during quiet wakefulness and upon whisking (Fig. 6*C*), suggesting that the wTLM → wS1 signaling affects the network status of wS1. Analyzing firings of individual units, however, we did not observe significant changes in the overall baseline AP rate of wS1 units by the wTLM → wS1 inactivation during quiet wakefulness, compared to the control (Fig. 6*D*). We further analyzed the effect of photo-inhibition of the wTLM → wS1 axons on the whisking-related modulation of wS1 units (Fig. 6*E*). Upon photo-inhibition, 40% (10 out of 25) of W-Exc and 18.5% (12 out of 65) of W-Inh RS units lost their responsiveness to whisking (Fig. 6*F*,*G*). Consistently, the averaged W-ΔAP of W-Exc RS units was largely attenuated (Fig. 6*H*; Light OFF 1.22 ± 0.11, Light ON 0.79 ± 0.18, *n* = 25 units, *p* = 0.0074, Wilcoxon signed rank test) and that of W-Inh RS units was significantly increased (Fig. 6*H*; Light OFF −0.75 ± 0.04, Light ON −0.63 ± 0.04, *n* = 65 units, *p* = 0.046, Wilcoxon signed rank test). In FS units, the increase in whisking-related activity of W-Exc units was strongly suppressed by photo-inhibition (Fig. 6*H*; Light OFF 1.39 ± 0.18, Light ON 0.64 ± 0.25, *n* = 15 units, *p* = 0.0083, Wilcoxon signed rank test). However, the photo-inhibition did not affect W-Inh FS units (Fig. 6*H*; Light OFF −1.29 ± 0.09, Light ON −1.12 ± 0.07, *n* = 15 units, *p* = 0.21, Wilcoxon signed rank test). These results suggest that the thalamocortical axons well signal whisking to affect wS1 activity. These effects of photo-inhibition on RS and FS units are in good agreement with the previously proposed feed-forward inhibition circuits recruited by the thalamocortical inputs (Gabernet et al., 2005; Yu et al., 2016).

**Figure 7.**
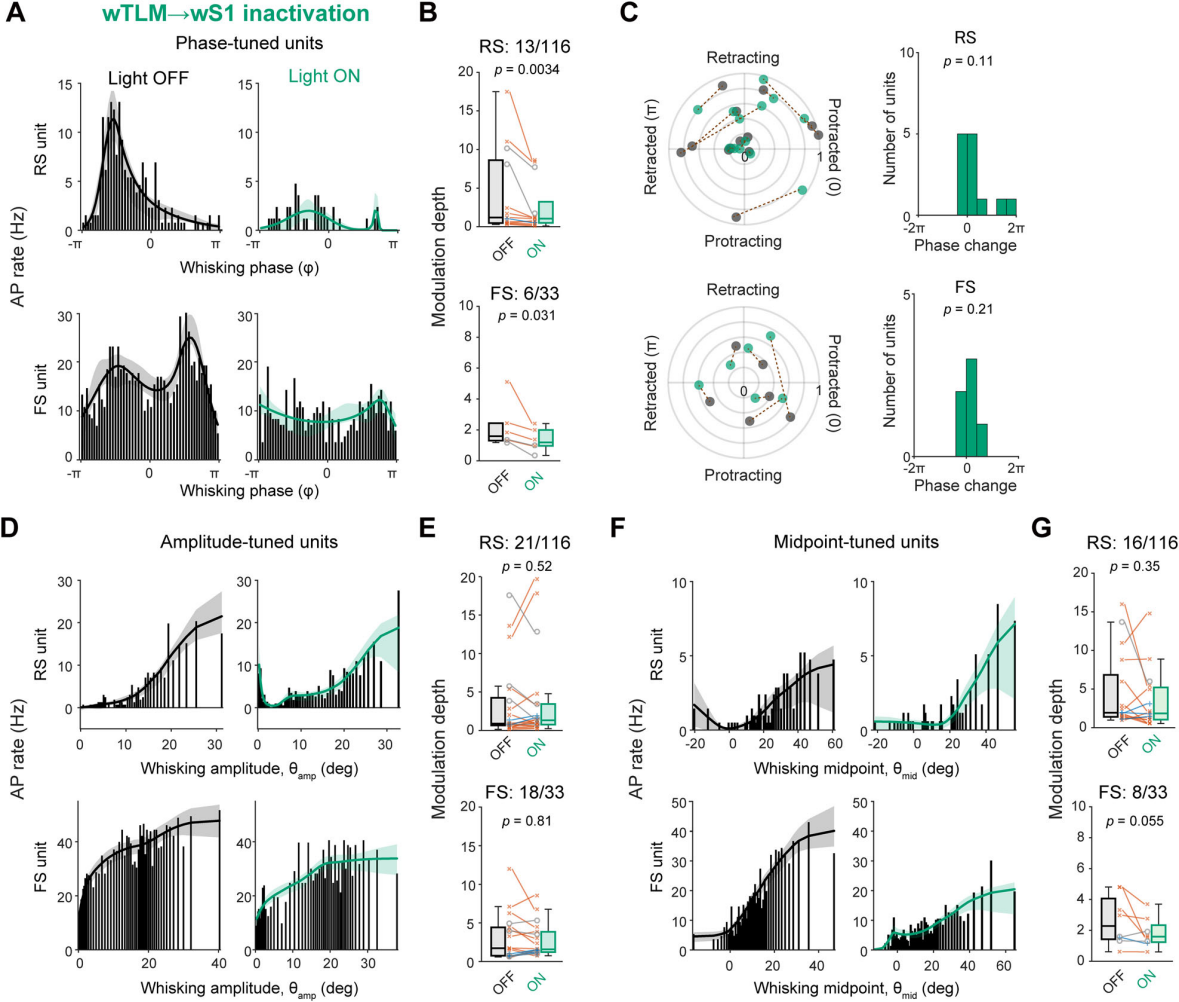
Specific involvement of wTLM → wS1 signaling in whisking phase tuning of wS1 neurons. ***A***, Histograms and tuning curves of representative RS (top) and FS (bottom) units tuned to whisking phase with (Light ON) and without (Light OFF) inactivation of wTLM → wS1 axons. ***B***, Modulation depths of phase-tuned units with (ON) and without (OFF) wTLM → wS1 inactivation. ***C***, Left, the whisking phase-locked modulation index and maximal-firing phase of individual phase-tuned units with (green) and without (black) wTLM → wS1 inactivation. Dotted lines connect data from identical units. Right, the distribution of shifts in the maximal-firing phase. ***D***, ***E***, Same as (***A***, ***B***), but for amplitude-tuned units. ***F***, ***G***, Same as (***A***, ***B***), but for midpoint-tuned units. Shadings in tuning curves (in ***A***, ***D***, ***F***) indicate 95% confidence intervals. The *p*-values are indicated in the figure panels. Wilcoxon signed rank test (***B*, *E*, *G***), or one-sample *t* test vs 0 (***C***).

We further analyzed the effects of the wTLM → wS1 photo-inhibition on the tuning of whisking-related variables in wS1 units. Strikingly, among the whisking phase-tuned units, the tuning magnitude was markedly attenuated by photo-inhibition (Fig. 7*A*,*B*; modulation depth: RS units, Light OFF 4.26 ± 1.48, Light ON 2.51 ± 0.88, *n* = 13 units, *p* = 0.0034, Wilcoxon signed rank test) without an overall trend of changing the maximal firing phase in the whisking cycle (Fig. 7*C*; shift of the maximal firing phase: 0.99 ± 0.55 rad, *n* = 13 units, *p* = 0.11, one sample *t* test vs 0). However, the photo-inhibition did not affect the tuning of θ_amp_- or θ_mid_-tuned units (Fig. 7*D*–*G*). The photo-inhibition also attenuated whisking phase-locked spike modulation in FS units (Fig. 7*A*,*B*; modulation depth: Light OFF 2.20 ± 0.56, Light ON 1.35 ± 0.28, *n* = 6 units, *p* = 0.031, Wilcoxon signed rank test) without changing the maximal firing phase (Fig. 7*C*; shift of the maximal firing phase: 0.49 ± 0.31 rad, *n* = 6 units, *p* = 0.21, one sample *t* test vs 0). Similarly to RS units, the modulation depths of θ_amp_- or θ_mid_-tuned FS units were not significantly affected by the photo-inhibition (Fig. 7*D*–*G*). These results suggest that thalamocortical inputs are critically involved in generating the whisking phase tuning, but not θ_amp_ and θ_mid_ tuning, in wS1 neurons.

**Figure 8.**
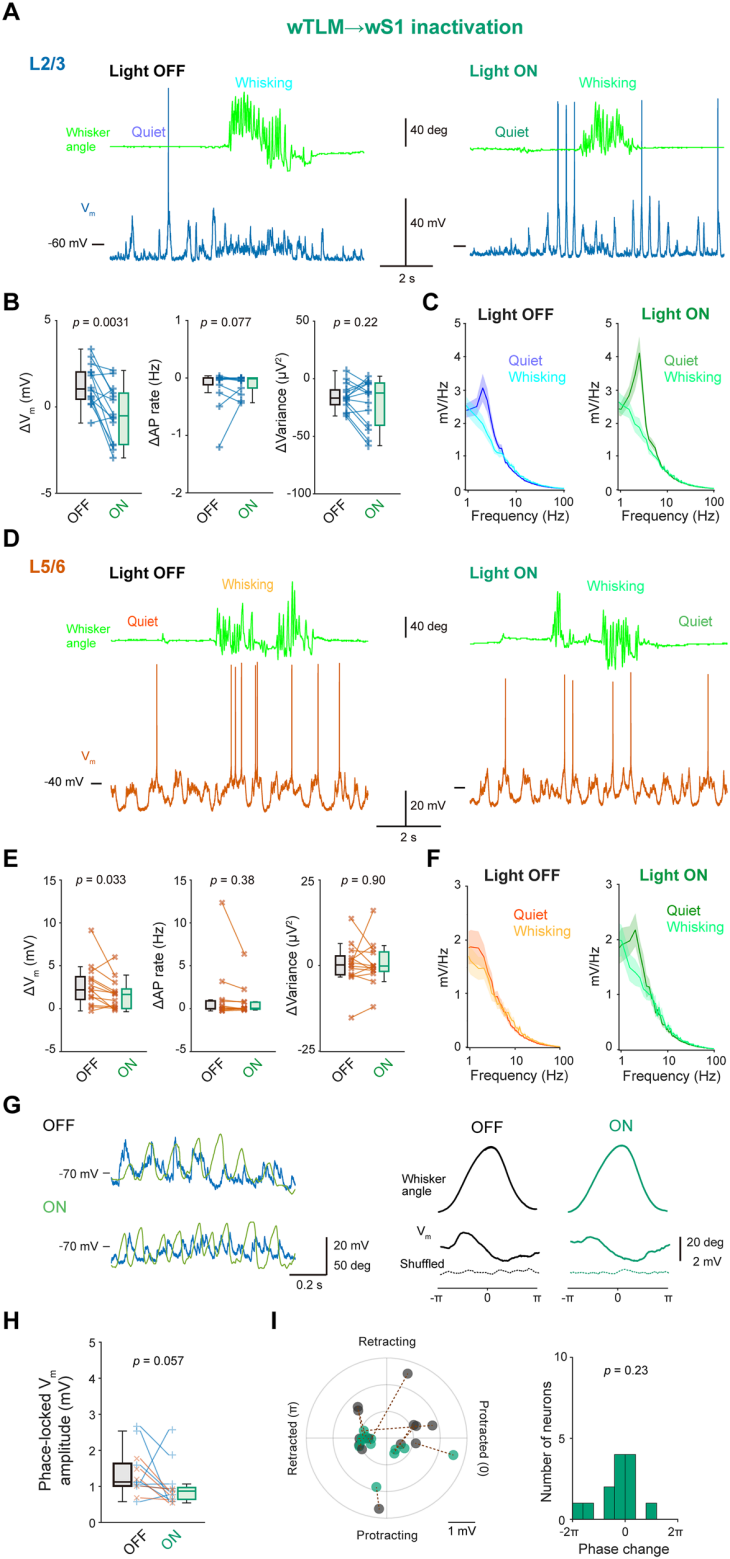
Effects of wTLM → wS1 inactivation on the membrane potential of wS1 neurons. ***A***, Example *V_m_* recording from a neuron at L2/3 of wS1 with (Light ON) and without (Light OFF) inactivation of the wTLM → wS1 axons. ***B***, Whisking-induced changes in mean *V_m_* (left), AP rate (middle), and *V_m_* variance (right) of L2/3 wS1 neurons with (ON) and without (OFF) wTLM → wS1 inactivation. ***C***, Averaged *V_m_* FFT during quiet wakefulness and whisking (*n* = 13 cells). ***D***–***F***, Same as (***A***–***C***), but from L5/6 neurons of wS1 (*n* = 14 cells). ***G***, Left, example *V_m_* recording during whisking from an L2/3 neuron in wS1 with (ON) and without (OFF) wTLM → wS1 inactivation. Right, averaged whisker angle (top), averaged *V_m_* traces (middle), and shuffled *V_m_* averages (bottom) as a function of whisking phase, obtained from the example neuron. ***H***, Amplitudes of whisking phase-locked *V_m_* fluctuation with (ON) and without (OFF) inactivation of wTLM → wS1 axons. ***I***, Left, the amplitude of phase-locked *V_m_* fluctuation and the most depolarized phase with (green) and without (black) wTLM → wS1 inactivation. Dashed lines connect identical cells. Right, distribution of the changes in the most depolarized phase. Solid line and shading (in ***C, F***) represent mean ± SEM. The *p*-values are indicated in the figure panels. Wilcoxon signed rank test (***B***, ***E*, *H***), one-sample *t* test vs 0 (***I***).

We next performed in vivo whole-cell recordings and examined how sub-threshold *V_m_* dynamics of wS1 neurons can be affected by photo-inhibition of the wTLM → wS1 axons (Fig. 8). In our recordings, the photo-inhibition depolarized the resting *V_m_* of wS1 neurons without affecting baseline AP rate during quiet wakefulness (Table 2). The photo-inhibition also attenuated *V_m_* depolarization upon whisking in both superficial and deep layers of wS1 (Fig. 8*A*,*B*,*D*,*E*; L2/3: Light OFF 1.3 ± 0.3 mV, Light ON −0.4 ± 0.4 mV, *n* = 14 cells, *p* = 0.0031, Wilcoxon signed rank test; L5/6: Light OFF 2.7 ± 0.7 mV, Light ON 1.7 ± 0.5 mV, *n* = 13 cells, *p* = 0.033, Wilcoxon signed rank test) without affecting whisking-related changes in AP rates and *V_m_* variance (Fig. 8*B,E*). The photo-inhibition also slightly enhanced slow-wave *V_m_* oscillation during quiet wakefulness (FFT area at 1−5 Hz, Light OFF 6.3 ± 0.6 mV, Light OFF 8.5 ± 0.9 mV, *n* = 27 cells, *p* = 0.030, Wilcoxon signed rank test; Fig. 8*C*,*F*). All these effects are similar to those previously reported after the pharmacological inhibition of wTLM (Poulet et al., 2012). A subset of wS1 neurons showed whisking phase-locked *V_m_* modulation, and their modulation depths tended to be slightly attenuated by the photo-inhibition, although its effect was not statistically significant (Fig. 8*G*–*I*; Light OFF 1.4 ± 0.2 mV, Light ON 1.0 ± 0.2 mV, *n* = 13, *p* = 0.057). These results suggest that a whisking-induced increase in wTLM activities affects wS1 neurons through direct thalamocortical connections.

### Somatosensory feedback shapes wS1 activity in quiet and whisking states

**Figure 9.**
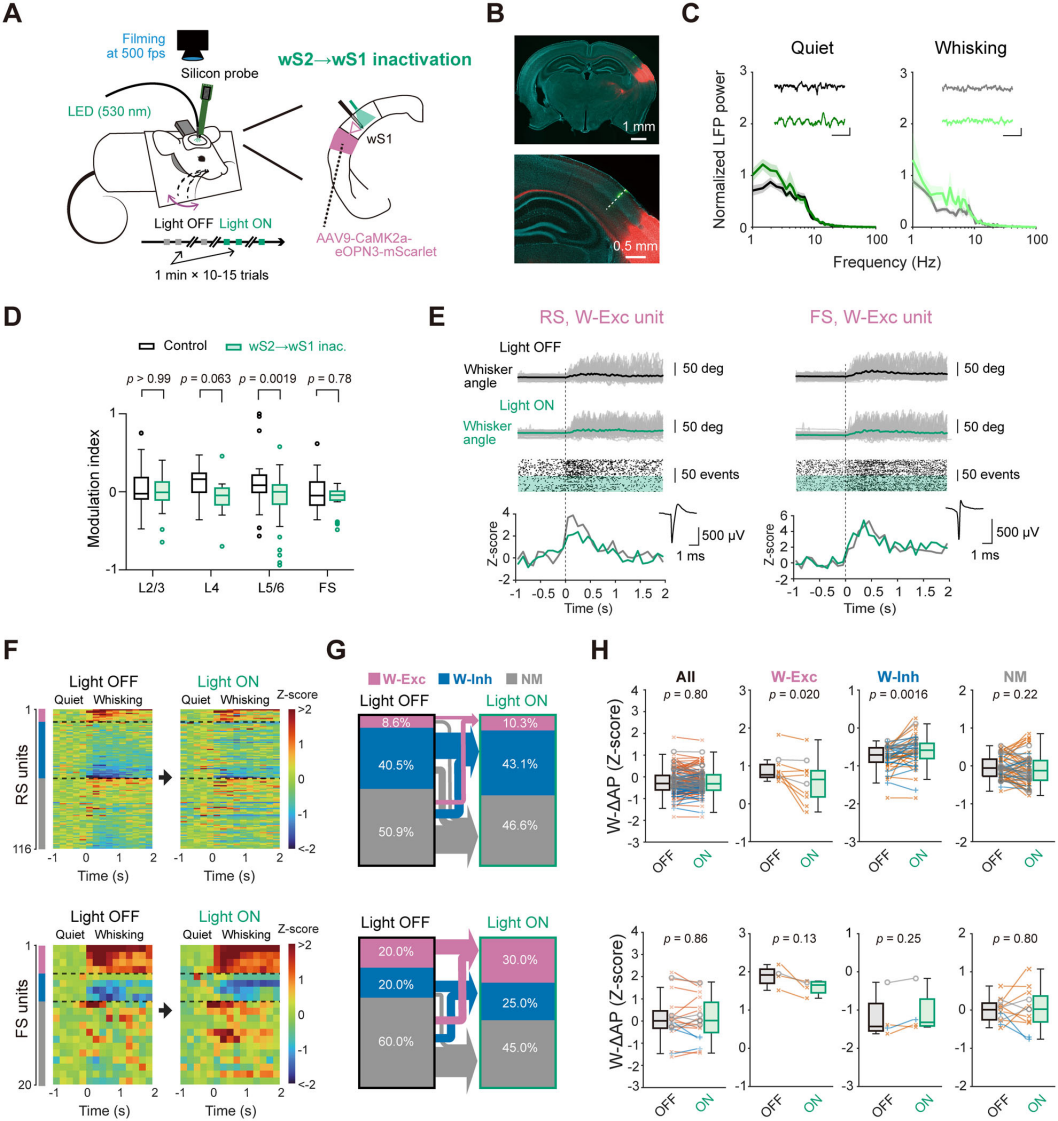
Effects of inactivation of wS2 → wS1 axons on neuronal activity in wS1. ***A***, Schematic of the experiment. ***B***, Epifluorescence image of a coronal brain section containing the wS2 injection site and the wS1 recording site obtained with a lower (top) and higher (bottom) magnification. The dashed line indicates the inserted electrode’s trace. Green: DiO, red: eOPN3-mScarlet, blue: DAPI. ***C****,* Normalized LFP power spectra in wS1 during quiet wakefulness (left) and whisking (right), with (green or light green) or without (black or gray) inactivation of wS2 → wS1 axons. Insets: representative LFP traces with (bottom) and without (top) inactivation. The inactivation modulated the slow components of LFP signals (1−5 Hz power; quiet: *F*_(1,55)_ = 9.50, *p* = 0.0029; whisking: *F*_(1,55)_ = 8.08, *p* = 0.0060; two-way ANOVA, Light ON vs Light OFF, *n* = 4 mice). Scale bar: 0.5 s, 0.2 mV; solid line and shading: mean ± SEM. ***D***, Modulation indices of baseline AP rates during quiet wakefulness upon green light illumination onto wS1 in control mice (same data as in Fig. 2*F*) or eOPN3-expressing mice (wS2 → wS1 inac., 136 units from 3 mice). Open circles indicate data points from outliers. ***E***, Example whisker angle timeplots (top), raster plots (middle), and *Z*-scored PETH (bottom) aligned to the whisking onset with (Light ON) or without (Light OFF) inhibition of wS2 → wS1 axons from representative RS (left) and FS (right) units. Small insets on the bottom show averaged AP waveforms of the corresponding units. Green shading in the raster plot indicates Light ON trials. ***F***, The *Z*-scored PETHs of individual RS (top) and FS (bottom) units (200 ms bin size) aligned to the whisking onset with (Light ON) and without (Light OFF) inactivation of the wS2 → wS1 axons. ***G***, Proportions of whisking-modulated RS (top) or FS (bottom) units in wS1 with (Light ON) and without (Light OFF) the wS2 → wS1 inactivation, separated into the W-Exc, W-Inh, and NM categories. Arrows show transitions of the categories. ***H***, W-ΔAP (in *Z*-score) of RS (top) and FS (bottom) units with (Light ON) and without (Light OFF) the wS2 → wS1 inactivation. Blue lines and crosses: L2/3 units, gray lines and circles: L4 units, orange lines and saltires: L5/6 units. The *p*-values are indicated in the figure panels. Bonferroni’s multiple comparison test (***D***), ****Wilcoxon signed rank test (***H***, except for FS All units), or paired *t* test (***H***, FS All units).

The wS2 has a prominent motor-related activity during task performance (Chen et al., 2016; Matteucci et al., 2022), which can be signaled to wS1 through direct feedback connections (Yang et al., 2016; Minamisawa et al., 2018). We next examine the role of wS2 → wS1 inputs in driving the whisking-related modulation of wS1 neurons. We locally administered AAV to induce expression of eOPN3 in wS2 neurons and subsequently conducted silicon probe recordings from the C2 barrel column of ipsilateral wS1 (Fig. 9*A,B*). Our analysis of the LFP power at L5 showed an enhancement of the slow components at 1−5 Hz upon photo-inhibition of the wS2 → wS1 axons (Fig. 9*C*), suggesting an effect on the network status of wS1. During quiet wakefulness, photo-inhibition of the wS2 → wS1 input significantly inhibited overall baseline AP rates of infragranular (L5/6) RS units [modulation index: mCherry (control) 0.12 ± 0.05, *n *= 36 units, eOPN3 −0.080 ± 0.036, *n* = 65 units, *p* = 0.0019, Bonferroni’s multiple comparisons test] but did not affect those of RS units in other layers and FS units (Fig. 9*D*). Thus, the baseline signaling from wS2 to wS1 may activate wS1 neurons in deeper layers during non-whisking, quiet states.

**Figure 10.**
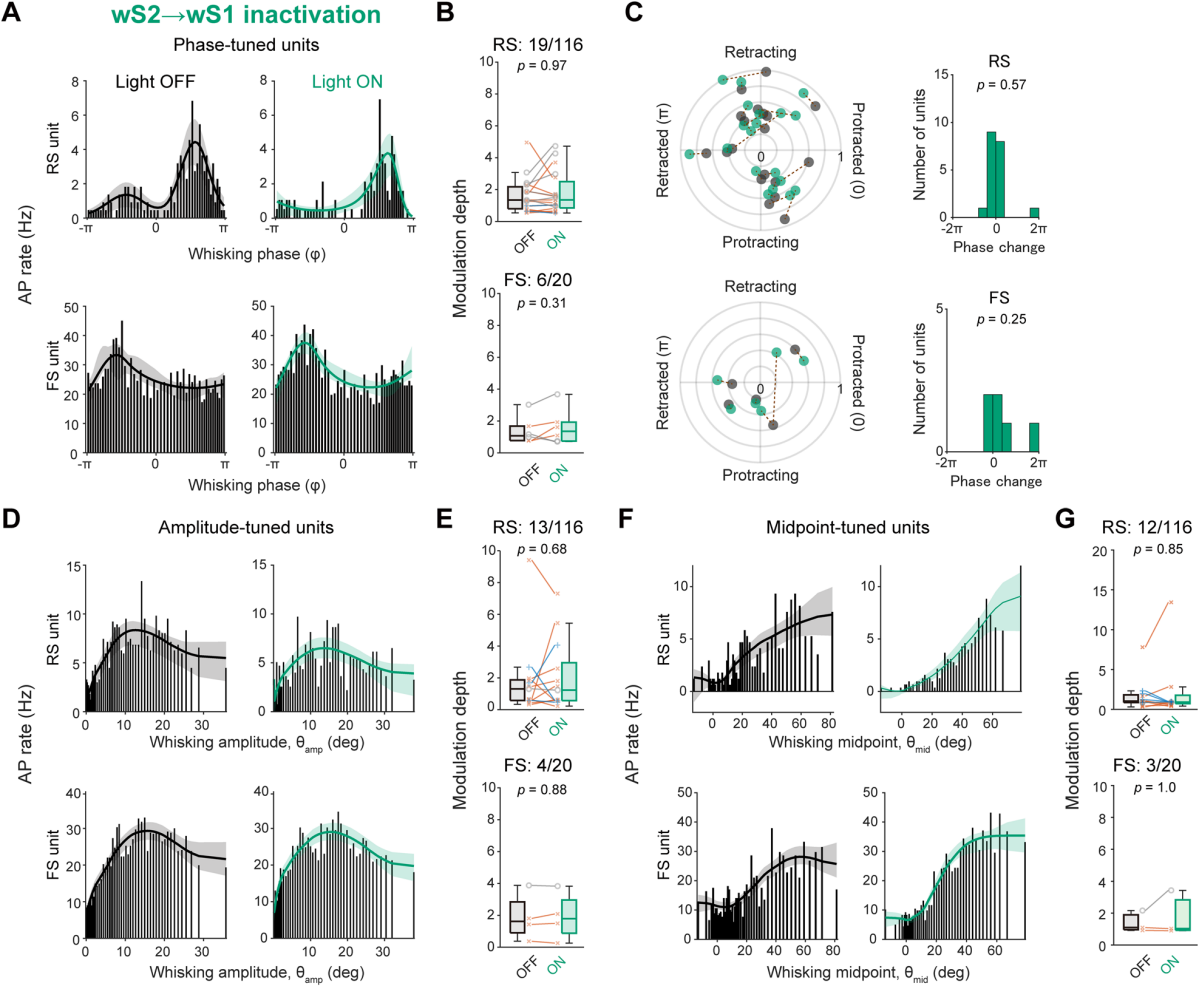
No involvement of wS2 → wS1 signaling in whisking variable tuning of wS1 neurons. ***A***, Histograms and tuning curves of representative RS (top) and FS (bottom) units tuned to whisking phase with (Light ON) and without (Light OFF) inactivation of wS2 → wS1 axons. ***B***, Modulation depths of phase-tuned units with (ON) and without (OFF) wS2 → wS1 inactivation. ***C***, Left, the whisking phase-locked modulation index and maximal-firing phase of individual phase-tuned units with (Light ON) and without (Light OFF) the wS2 → wS1 inactivation. Dotted lines connect data from identical units. Right, the distribution of shifts in the maximal-firing phase. ***D***, ***E***, Same as (***A***, ***B***), but for amplitude-tuned units. ***F***, ***G***, Same as (***A***, ***B***), but for midpoint-tuned units. Shadings in tuning curves in (***A***, ***D***, ***F***) indicate 95% confidence intervals. The *p*-values are indicated in the figure panels. Two-sample (***B***, ***E***, ***G***) or one-sample (RS units in ***C*** vs 0) Wilcoxon signed rank test, or one-sample *t* test vs 0 (FS units in ***C***).

We further analyzed the effect of photo-inhibition of the wS2 → wS1 axons on the whisking-related modulation of wS1 units (Fig. 9*E*). Upon photo-inhibition, 50.0% (5 out of 10) of W-Exc and 21.3% (10 out of 37) of W-Inh RS units lost their responsiveness to whisking (Fig. 9*F*,*G*). Overall, the whisking-induced changes in AP rates of W-Exc and W-Inh RS units were significantly attenuated by the wS2 → wS1 photo-inhibition (Fig. 9*H*; W-ΔAP; W-Exc: Light OFF 0.91 ± 0.11, Light ON 0.60 ± 0.17, *n *= 10 units, *p* = 0.020, Wilcoxon signed rank test; W-Inh: Light OFF −0.76 ± 0.05, Light ON −0.61 ± 0.06, *n* = 47 units, *p* = 0.0016, Wilcoxon signed rank test). In contrast, the photo-inhibition did not significantly affect the whisking-related modulation of FS units in wS1 (Fig. 9*F–H*). Thus, the wS2 → wS1 inputs are involved in the whisking-related modulation of the neuronal activities in wS1. Nevertheless, the wS2 → wS1 inactivation failed to influence the tuning magnitudes in the wS1 units that exhibit a significant tuning to the whisking variables (Fig. 10). These results suggest that wS2 → wS1 inputs may contribute to the excitation of wS1 cells in response to whisker motion, irrespective of the position of whiskers.

**Figure 11.**
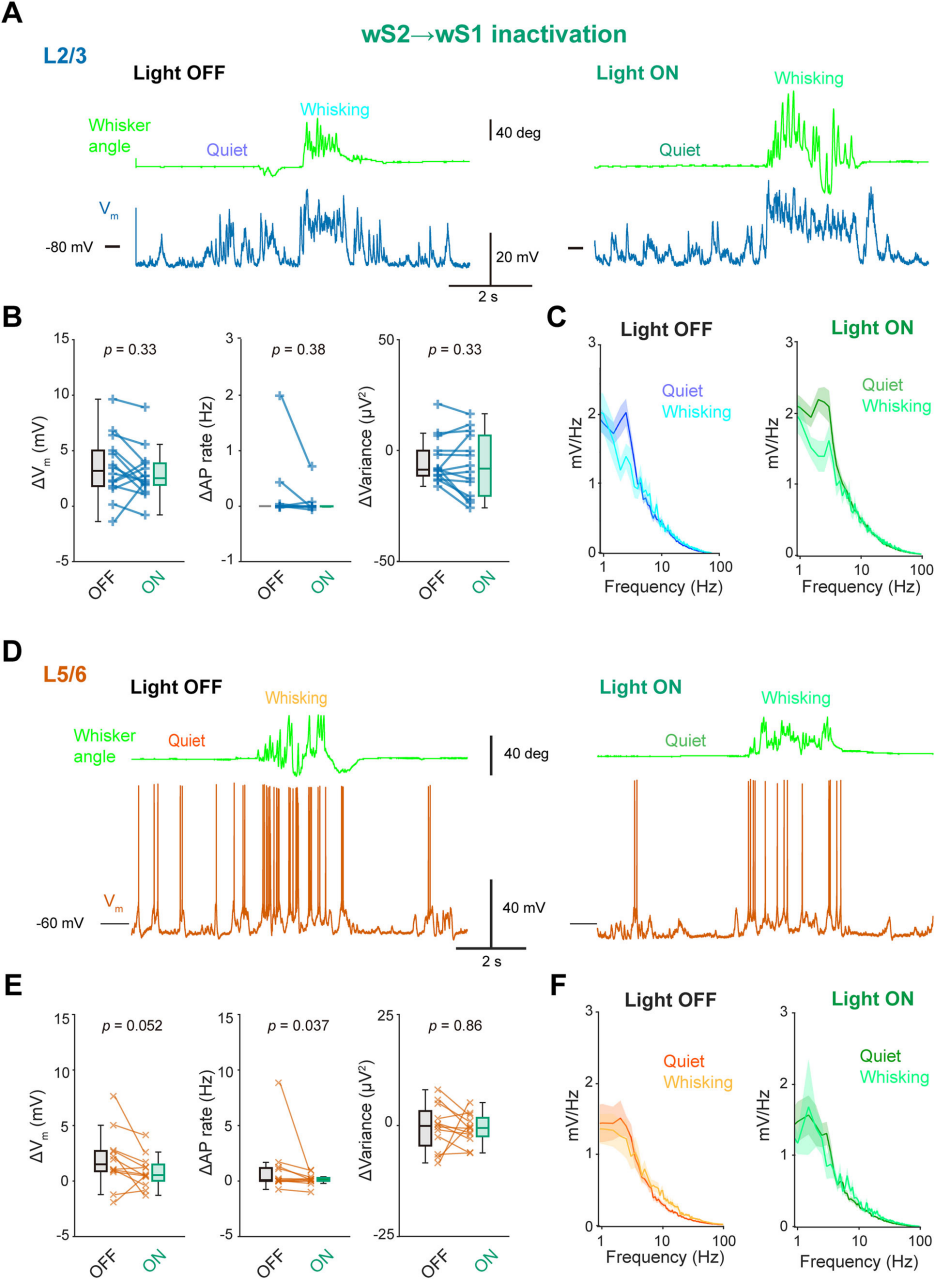
Effects of wS2 → wS1 inactivation on the membrane potential of wS1 neurons. ***A***, Example *V_m_* recording from a neuron at L2/3 of wS1 with (Light ON) and without (Light OFF) inactivation of wS2 → wS1 axons. ***B***, Whisking-induced changes in mean *V_m_* (left), AP rate (middle), and *V_m_* variance (right) of L2/3 wS1 neurons with (ON) and without (OFF) wS2 → wS1 inactivation. ***C***, Averaged *V_m_* FFT during quiet wakefulness and whisking (*n *= 12 cells). ***D***–***F***, Same as (***A***–***C***), but from L5/6 neurons of wS1 (*n* = 10 cells). Solid line and shading (in ***C, F***) represent mean ± SEM. The *p*-values are indicated in the figure panels. Wilcoxon signed rank test (***B***, ***E***)

**Figure 12.**
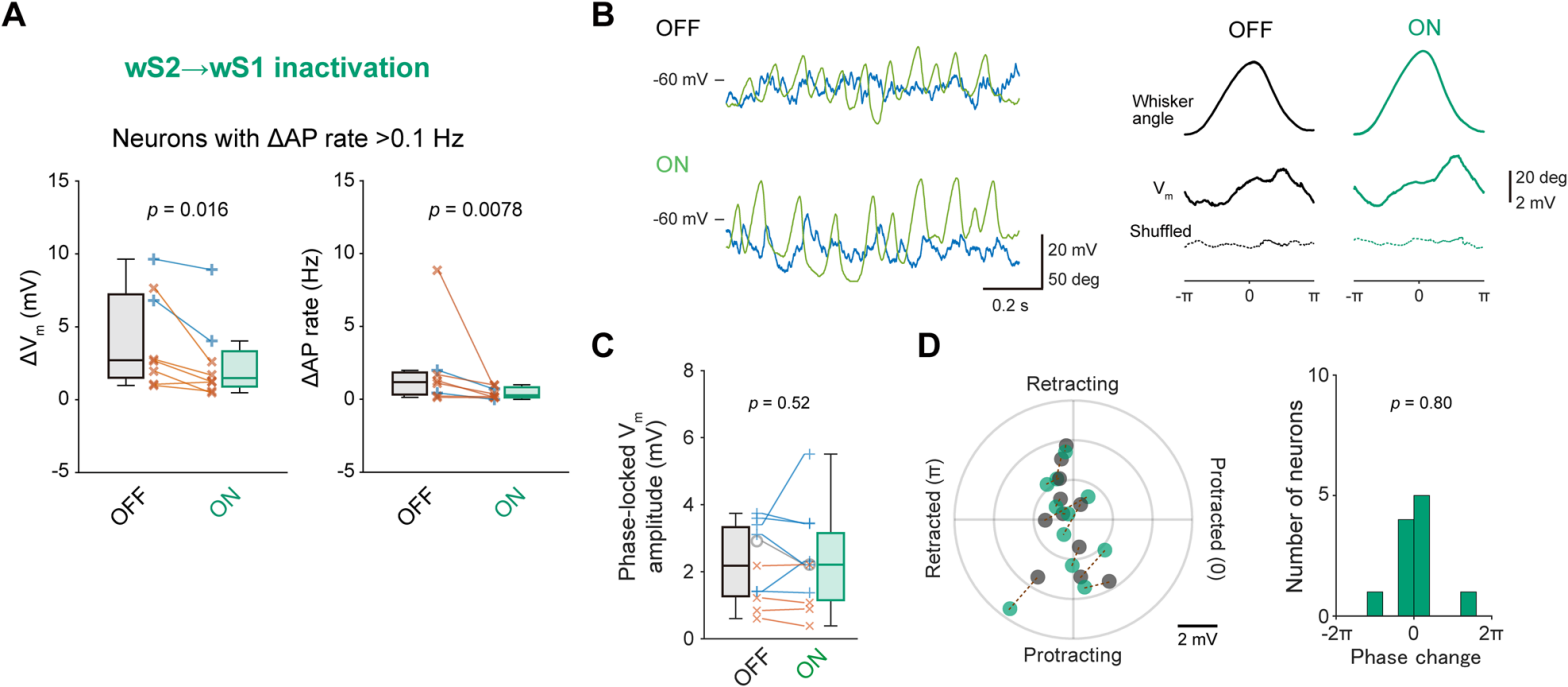
The wS2 → wS1 signaling depolarizes a subset of wS2 neurons during whisking without providing information on the whisker position. ***A***, Whisking-induced changes in mean *V_m_* (left) and AP rate (right) of wS1 neurons that showed a whisking-induced increase of AP rate by more than 0.1 Hz, with (Light ON) and without (Light OFF) wS2 → wS1 inactivation. Lines indicate individual data from L2/3 (blue) or L5/6 (orange). ***B***, Left, example *V_m_* recording during whisking from an L2/3 neuron in wS1 with (ON) and without (OFF) wS2 → wS1 inactivation. Right, averaged whisker angle (top), averaged *V_m_* traces (middle), and shuffled *V_m_* averages (bottom) as a function of whisking phase, obtained from the example neuron. ***C***, Amplitudes of whisking phase-locked *V_m_* fluctuation with (ON) and without (OFF) inactivation of wS2 → wS1 axons. ***D***, Left, the amplitude of phase-locked *V_m_* fluctuation and the most depolarized phase with (green) and without (black) wS2 → wS1 inactivation. Dashed lines connect identical cells. Right, distribution of the changes in the most depolarized phase. The *p*-values are indicated in the figure panels. Wilcoxon signed rank test (***A***), or one-sample *t* test vs 0 (***D***).

We also performed whole-cell patch-clamp recordings from wS1 neurons to examine the effects of the wS2 → wS1 photo-inhibition on *V_m_* dynamics. At L2/3, the photo-inhibition did not affect the resting *V_m_* and baseline AP rates (Table 2), and it also did not influence whisking-induced changes in *V_m_*, AP rate, *V_m_* variance, and *V_m_* fluctuation in neurons (Fig. 11*A*–*C*). At L5/6, although the silicone probe data suggested a significant contribution of the wS2 → wS1 signaling to the baseline AP rate, the photo-inhibition of wS2 → wS1 axons failed to affect the resting *V_m_* and baseline AP rates (Table 2), possibly due to the inclusion of neurons with lower AP rates. However, the photo-inhibition apparently reduced the whisking-induced AP rate changes at L5/6 (Light OFF 1.0 ± 0.7 Hz, Light ON 0.1 ± 0.1 Hz, *n* = 12 cells, *p* = 0.037; Fig. 11D,*E*), but its effect on the whisking-induced *V_m_* depolarization, *V_m_* variance, and slow-wave *V_m_* fluctuation was not evident (Fig. 11*E*,*F*). In eight neurons that exhibited an increase in AP rates by more than 0.1 Hz during whisking, significant suppression of whisking-related depolarization and spiking was observed with the wS2 → wS1 photo-inhibition (Fig. 12*A*,*B*; *V_m_* depolarization: Light OFF, 4.2 ± 1.1 mV, Light ON, 2.6 ± 0.9 mV, *p* = 0.016, Wilcoxon signed rank test; ΔAP rate: Light OFF, 2.0 ± 0.9 Hz, Light ON, 0.4 ± 0.1 Hz, *p* = 0.0078, Wilcoxon signed rank test). Our results thus indicate that the somatosensory feedback connections from wS2 to wS1 depolarize a subset of wS1 neurons during whisking and generate the whisking-related activity patterns of these neurons. Despite such modulation, the wS2 → wS1 photo-inhibition did not affect the magnitude of whisking phase-locked *V_m_* modulation in neurons with significant phase-locking to the whisking cycle (Fig. 12*C*–*E*; Light OFF 2.2 ± 0.4 mV, Light ON 2.3 ± 0.4 mV, *n* = 11 cells, *p* = 0.52), consistent with the results from extracellular recordings showing no impact of wS2 → wS1 inputs on whisking variable tuning in wS1 (Fig. 10).

### Whisker motion characteristics during photo-inhibition

**Figure 13.**
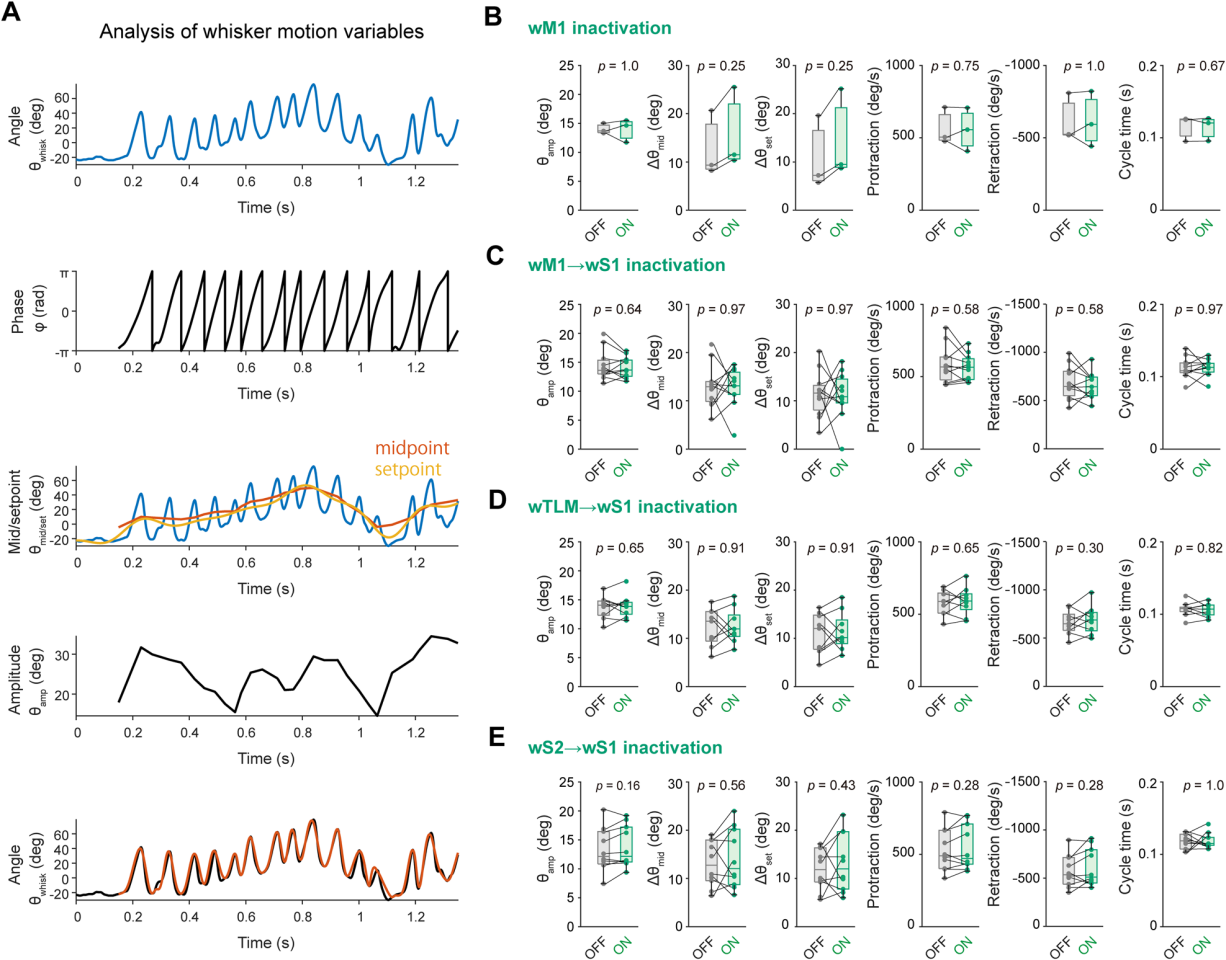
No effect of optogenetic inhibition of wM1 and wM1/wTLM/wS2 → wS1 signaling on whisker motion characteristics. ***A***, Example recording of a whisking bout (top) and its decomposition using Hilbert transformation. Whisking phase, midpoint, setpoint, and amplitude were extracted. The position of the right C2 whisker was accurately reconstructed using these variables (bottom, orange). ***B***, Averages of whisking amplitude (θ_amp_), midpoint amplitude (Δθ_mid_), setpoint amplitude (Δθ_set_), protraction speed, retraction speed, and whisking cycle duration (cycle time) with (ON) and without (OFF) inactivation of left wM1. Lines indicate individual mouse data (*n* = 3 mice). ***C***, Same as ***B***, but for wM1 → wS1 inactivation (*n* = 11 mice). ***D***, Same as ***B***, but for wTLM → wS1 inactivation (*n* = 9 mice). ***E***, Same as ***B***, but for wS2 → wS1 inactivation. The *p*-values are indicated in the figure panels. Wilcoxon signed rank test (***B***, ***C***, ***D***, ***E***, except for cycle time in ***B***) or paired *t* test (cycle time in ***B***).

The present study clarified the role of interareal synaptic inputs for the generation of whisking-related supra- and sub-threshold *V_m_* dynamics of wS1 neurons. It is important to examine whether our optogenetic manipulation applied to the circuits could introduce any confounding effects through changing whisking behavior. To address this issue, we analyzed the effect of our circuit intervention on whisker motion characteristics. Using the Hilbert transform, we extracted the various parameters related to whisking behavior, such as amplitude, setpoint, midpoint, protraction/retraction speed, and whisking cycle duration (Fig. 13). Despite a range of effects on whisking-related activity in wS1 upon circuit perturbation, none of these manipulations significantly affected any of these whisker kinematics parameters. Thus, our conclusions based on electrophysiological experiments were not confounded by alterations in whisking behavior.

## Discussion

Using eOPN3-mediated photo-inhibition of synaptic transmission, we investigated the role of interareal synaptic inputs to wS1 in the whisking-related modulation of supra- and sub-threshold *V_m_* of wS1 neurons. We found that wTLM → wS1 and wS2 → wS1 projections significantly shape the whisking-related activity patterns within wS1 by affecting the neuronal *V_m_* dynamics. In contrast, wM1 → wS1 projections appear to have little overall influence on the whisking-related modulation of wS1 neurons.

Neurons in wM1 represent various aspects of whisker movements (Hill et al., 2011; Friedman et al., 2012; Gerdjikov et al., 2013; Sreenivasan et al., 2016), and the stimulation of wM1 neurons initiates rhythmic whisking (Ferezou et al., 2007; Matyas et al., 2010; Sreenivasan et al., 2015, 2016; Li et al., 2023). The wM1 neurons have extensive axonal projections to L1 and L5 at wS1, with monosynaptic connections to wS1 neurons (Petreanu et al., 2009; Kinnischtzke et al., 2014). Optogenetic stimulation of wM1 axons in wS1 *in vivo* causes state changes in wS1 neurons, similar to whisking-induced ones (Zagha et al., 2013). Among wS1 neurons, GABAergic neurons expressing vasoactive intestinal peptide-expressing (VIP) receive the most robust excitatory inputs from wM1 (Lee et al., 2013; Naskar et al., 2021). Excitation of VIP neurons is known to disinhibit nearby excitatory pyramidal neurons, mostly at distal dendrites, by inhibiting somatostatin (SST)-expressing GABAergic neurons (Lee et al., 2013; Pfeffer et al., 2013; Pi et al., 2013; Zhang et al., 2014). Moreover, regional inhibition of wM1 causes insensitivity of VIP and SST neurons in wS1 to whisking (Lee et al., 2013) and largely attenuates dendritic touch responses of wS1 L5 neurons (Xu et al., 2012). Therefore, it has long been hypothesized that wM1 neurons would affect wS1 neurons through exciting VIP neurons during whisking, recruiting the disinhibitory circuit.

Photo-inhibition using eOPN3 proved effective across all cortical layers (Fig. 1). Our LFP analysis in wS1 revealed a significant effect of inhibiting wM1 → wS1 signaling (Fig. 2*C*), consistent with a previous report (Zagha et al., 2013). We also observed various notable effects of inhibiting axons from wTLM or wS2 in wS1. These results validate the efficacy of our experimental manipulations. Nevertheless, our results provide little evidence of the role of wM1 → wS1 inputs in whisking-induced modulation of the AP rates and *V_m_* dynamics of wS1 neurons. Thus, any modulatory influence the wM1 → wS1 pathway may have on wS1 is subtle, potentially falling below the detection threshold of our current experimental settings – the neurons projecting from wM1 to wS1 might exhibit low firing rates during whisking. In future studies, the employment of more potent opsins, such as the recently developed potassium-selective channelrhodopsins (Govorunova et al., 2022; Vierock et al., 2022; Tajima et al., 2023), could offer a stronger inhibitory effect than eOPN3 and may therefore reveal more nuanced aspects of how the wM1 → wS1 pathway influences wS1 activity.

In the primary auditory cortex (A1), like in wS1, optogenetic stimulation of the top-down projections from the frontal motor cortex to A1 induces depolarization and the reduction of slow-wave *V_m_* oscillation in A1 neurons as seen upon body movements (Schneider et al., 2014). The disinhibitory VIP-SST circuit exists in A1 as in wS1 (Pi et al., 2013). However, the disinhibitory mechanism seems not to operate for the motor-related modulation of A1 neurons (Yavorska and Wehr, 2021). The primary visual cortex (V1) also implicates a similar VIP-SST disinhibitory circuit, which is recruited mainly by cholinergic inputs during locomotion (Fu et al., 2014). In wS1, cholinergic axons from the basal forebrain are activated during whisking and release acetylcholine to excite VIP neurons (Eggermann et al., 2014; Gasselin et al., 2021). Taken together, the top-down wM1 → wS1 inputs might not be the primary mechanism for generating whisking-induced state changes in wS1 neurons. Rather, cholinergic inputs may have a major role. However, given that our movement analysis was confined solely to whisker movements, we cannot rule out the possibility that movements in other body parts could have occurred and potentially influenced the neural activity recorded in our study. Future investigations might require exploring the interactions between neuromodulatory systems and whisker motor-sensory inputs and their potential role in state-dependent modulation and sensory processing, in combination with thorough measurements of body motions.

During episodes of whisking, neurons within the wTLM receive excitatory synaptic inputs and enhance their firing rates (Moore et al., 2015; Urbain et al., 2015). Our data illustrate that inactivation of the wTLM → wS1 projections amplifies slow-wave *V_m_* oscillations and decreases the depolarization of wS1 neurons during whisking. These observations echo those made in a previous study that employed regional wTLM inhibition (Poulet et al., 2012), supporting a hypothesis that wTLM’s role in the whisking-related state changes within wS1 is, at least partially, mediated through direct synaptic inputs to wS1. Our results also revealed the critical role of wTLM → wS1 projections in generating the whisking phase tuning in wS1 (Fig. 7). A subset of neurons residing in the ventral posterior medial nucleus (VPM) of wTLM fires spikes phase-locked to the whisking cycle during whisking (Moore et al., 2015; Urbain et al., 2015). The wTLM receives inputs from various sources, including the trigeminal complex, which contains neurons showing whisking phase-locked spiking (Leiser and Moxon, 2007; Moore et al., 2015). Thus, sensory reafference, causing the neuronal tuning of the whisking phase through the whisker sensory relay pathway, is likely the primary driver of the whisking phase tuning in wS1. On top of the whisking phase, synaptic inputs to wTLM could provide a rich context for motor-related information (Simons, 1985; Urbain et al., 2015; Bellavance et al., 2017). Therefore, wTLM could substantially integrate diverse sensory and motor-related inputs, forwarding processed information to wS1. Thalamocortical inputs from VPM primarily terminate to wS1 L4, where the direct effect of thalamocortical inputs could be observed. However, the limited number of neurons recorded from this layer in our current dataset constrained our capacity for an extensive analysis of the cellular activity in L4. Therefore, in future studies, it would be of great interest to examine how the direct thalamocortical signals affect the activities of L4 neurons during sensorimotor integration.

Our results highlight the potential influence of wS2 → wS1 feedback signaling in modulating wS1 activity during whisking. The effect of the photo-inhibition of wS2 → wS1 inputs on baseline AP rates and whisking-related depolarization was primarily observable in the deeper layers (L5/6) of wS1. This result aligns with anatomical evidence showing more extensive innervation of wS2 axons within these deeper layers than L2/3 (Minamisawa et al., 2018; Zhang and Bruno, 2019). Given that wS2 receives excitatory synaptic inputs from wTLM, it is plausible that wS2 serves as a relay for whisking-related wTLM activity toward wS1 (El-Boustani et al., 2020). In contrast to the prominent role of wTLM → wS1 signaling in whisking phase encoding in wS1, however, the inactivation of wS2 → wS1 axons did not affect the neuronal representation of whisking variables in wS1 (Fig. 10), suggesting that this pathway signals whisking motion states rather than the detailed information of whisker position.

wS2 receives top-down inputs from the secondary motor cortex, which may play a critical role in transmitting motor-related signals to wS1 via wS2 (Matteucci et al., 2022). In the visual cortices, locomotion and eye movements enhance sensory responses in secondary visual areas (V2), which have feedback connections to V1 (Andermann et al., 2011). Such motor-related signals may be derived from the monosynaptic projection from the frontal motor areas to V2 (Itokazu et al., 2018). Thus, motor-related signal transmission from the motor cortex toward the primary sensory cortex via the secondary sensory cortex may be a circuit motif for sensorimotor integration in multiple sensory modalities.

Our whole-cell recordings revealed at least part of the mechanisms underlying whisking-related depolarization of wS1 cells, accounting for the presence of W-Exc cells in wS1. However, while approximately half of the extracellularly recorded units were identified as W-Inh cells, the underlying cellular mechanisms remain unknown. Despite the activities of these W-Inh units seemingly being shaped by wTLM → wS1 and wS2 → wS1 projections, our whole-cell recordings failed to capture these mechanisms—potentially due to lower baseline AP rates and shorter recording duration. This reinforces the need for technological advancements to secure sufficient spontaneous whisking epochs for a reliable comparison of AP rates.

Although our analysis primarily focuses on wS1 activity during spontaneous behaviors, the role of specific signaling pathways to wS1 can be modulated in mice engaged in cognitive or sensorimotor tasks. For instance, following the learning of a whisker detection task, the wS1 neurons projecting to wS2 show sensitivity to goal-directed licking behavior and undergo depolarization upon licking (Yamashita and Petersen, 2016). In line with this, the neurons within wS2 have been observed to accurately encode goal-directed licking, suggesting a potential enhancement of wS2 → wS1 signaling during goal-directed movement (Matteucci et al., 2022). Furthermore, the wM1 neurons projecting to wS1 also encode a variety of task- and motor-related information (Petreanu et al., 2012). Future studies involving task-performing mice will thus be crucial in further elucidating the role of specific long-range inputs to wS1 in shaping neuronal activity in wS1 during task-related movements.
